# Au(III) complexes with tetradentate-cyclam-based ligands

**DOI:** 10.3762/bjoc.17.18

**Published:** 2021-01-19

**Authors:** Ann Christin Reiersølmoen, Thomas N Solvi, Anne Fiksdahl

**Affiliations:** 1 Department of Chemistry, Norwegian University of Science and Technology, Høgskoleringen 5, 7491, Trondheim, Norway

**Keywords:** Au(III), carboalkoxylation, coordination studies, cyclam derivatives, cyclopropanation

## Abstract

Chiral cyclam (1,4,8,11-tetraazacyclotetradecane) derivatives were synthesized stepwise from chiral mono-Boc-1,2-diamines and (dialkyl)malonyl dichloride via open diamide-bis(*N-*Boc-amino) intermediates (65–91%). Deprotection and ring closure with a second malonyl unit afforded the cyclam tetraamide precursors (80–95%). The new protocol allowed the preparation of the target cyclam derivatives (53–59%) by a final optimized hydride reduction. Both the open tetraamine intermediates and the cyclam derivatives successfully coordinated with AuCl_3_ to give moderate to excellent yields (50–96%) of the corresponding novel tetra-coordinated *N,N,N,N*-Au(III) complexes with alternating five- and six-membered chelate rings. The testing of the catalytic ability of the cyclam-based *N,N,N,N*-Au(III) complexes demonstrated high catalytic activity of some complexes in selected test reactions (full conversion in 1–24 h, 62–97% product yields).

## Introduction

The importance of gold for humankind dates long back, and gold is linked to the evolution of many parts of the society. Contrary to the general fascination and importance of gold, the potential as homogenous catalyst has been neglected, compared to a range of other transition metals. The utilization of gold in synthetic organic chemistry has become a topic of interest during the last decades, as evidenced by the increasing number of review articles published in this period [1–8]. Whereas both gold(I) and gold(III) are proven to be catalytic active forms of gold, gold(I) has so far, received main attention, likely due to the higher stability, as demonstrated by the development of a high number of gold(I)-catalyzed transformations and ligated gold(I) complexes, along with improved mechanistic understanding [9–15]. In contrast, gold(III) catalysis was for a long time mostly based on inorganic salts, such as AuCl_3_, AuBr_3_, or pyridine–AuCl_3_ and Pic–AuCl_2_. However, Au(III) complexes with various coordinated ligands are about to become more explored. Different from the linear coordination mode of gold(I), gold(III) forms square planar complexes. This allows for greater steric control around the reaction center by using polydentate ligands. An interesting group of ligands which may coordinate to all the four coordination sites of gold(III), are represented by polyamine ligands, such as cyclam (1,4,8,11-tetraazacyclotetradecane), cyclen (1,4,7,10-tetraazacyclododecane)), ethylenediamine and triethylenetetraamine derivatives. Studies of Au(III)-cyclam modified complexes have been limited to arylated [16] or polymer-bound cyclams for selective uptake of Au(III) from water [17] as well as X-ray crystal structures [18–21]. Simple [Au(III)–cyclam] complexes have been investigated for various biological properties [22–29]. Particularly, studies have focused on their potential in vitro anticancer properties [24,26,29], the activity against a *falciparum* strain [22], the in vitro DNA binding properties [25] and reactions with bovine serum albumin [27].

Cyclam is known as a tetraamino-macrocyclic ligand, which binds strongly to give complexes with many transition metal cations. While catalytic applications of square planar cyclam complexes are reported for metals, such as Ni [30–33], Cu [34], Fe [35], catalytic properties of cyclam coordinated gold(III) complexes are not known. Trigged by this knowledge gap, we wanted to develop new chiral cyclam coordinated gold(III) complexes. Additionally, these complexes were interesting for the evaluation of the catalytic effect of the Au(III) complex upon substitution of all coordinating halides by nitrogen donors. We hereby present the synthesis of chiral cyclam ligands and related polyamino compounds, along with Au(III) coordination studies and evaluation of the catalytic ability of the successfully obtained Au(III) complexes in two model reactions.

## Results and Discussion

### Synthesis of potential ligands

**Scheme 1:**
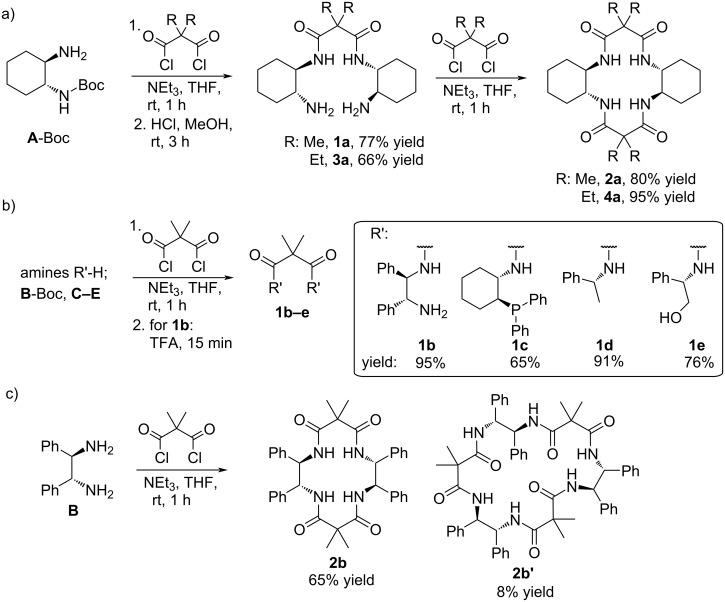
Synthetic protocols for the preparation of potential ligands **1**–**4**.

Chiral cyclam derivatives have previously been directly synthesized from (1*R*,2*R*)-cyclohexane-1,2-diamine (**A**) and malonyl dichloride [36], giving 36% yield of the wanted cyclam tetraamide product **2a**. Additionally, a macrocyclic byproduct (14%) was formed by condensation of three units of diamine **A** and malonyl dichloride. To inhibit the formation of the trimer, we decided to prepare the cyclams in an indirect way. In fact, increased yields of cyclam derivative **2a** (68% yield over three steps) were obtained by malonyl reaction of the mono-Boc-protected diamine (**A**-Boc) followed by Boc deprotection with HCl, and final ring closure of diamide–diamine intermediate **1a** with a second malonyl unit to give tetraamide product **2a** (Scheme 1a). The equivalent ethyl-substituted cyclam **4a** was prepared in comparable yield (63% over the three steps) by the same method with diethylmalonyl chloride. This method also allowed for isolation of the open diamide–diamine **1a** (77%). In addition, the similar potential ligands **1b–e** (65–95%, Scheme 1b) were likewise prepared from amines **B–E**. The phenyl-substituted cyclam tetraamide derivative **2b** was prepared by the original direct method [36] (65%, Scheme 1c), as the mono-Boc amine **B**-Boc was less accessible.

**Scheme 2:**
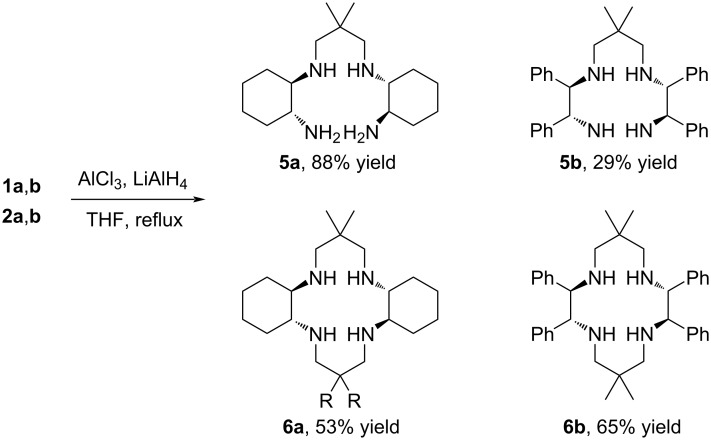
Reduction of diamides **1a**,**b** and tetraamides **2a**,**b**.

As the amide coordination to Au(III) in general is challenging, and not successful in our hands, as discussed below, we wanted to prepare the reduced amine products (**5a**,**b**, **6a**,**b**) from amides **1a**,**b** and **2a**,**b**. Initially, by refluxing diamide–diamines **1a**,**b** and cyclam amide precursors **2a**,**b** in THF with LiAlH_4_ for 3 days [36], complex product mixtures of partly and fully reduced species were obtained for all amides except **2a**. In order to activate the amides for reduction, improved reaction conditions were obtained by adding AlCl_3_ to the reactions. Complete reduction of polyamides **1a**,**b** and **2a**,**b** yielded the open tetraamine products **5a**,**b** and the target cyclams **6a**,**b** with four secondary amine functions in moderate to high yields (29–88%, Scheme 2) within 1–2 days.

### Au(III) coordination studies

Amide-coordinated Au(III) complexes have so far scarcely been reported [37–44]. This is likely a result of the electron deficient character of the amide nitrogens. The coordination was initially tested with the cyclam tetraamide derivatives **2a**,**b** and **4a**. Judged from ^1^H NMR, these ligands showed no interaction with Au(III), as expected. A similar resistance to coordinate was observed for the open diamides **1c–e**. The phosphorus containing ligand **1c** did undergo phosphorus oxidation instead of Au(III) coordination. No effect was obtained by refluxing or by adding additives, such as silver salts, NaOH or NH_4_PF_6_.

**Scheme 3:**
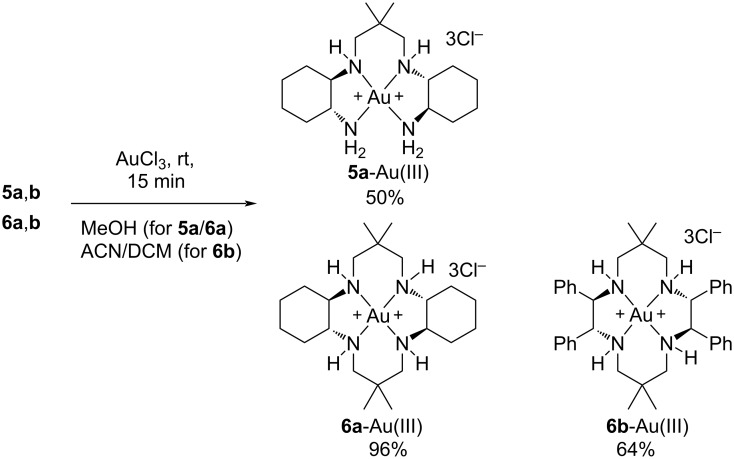
Au(III) coordination conditions for ligands **5a**,**b** and **6a**,**b**. Coordination of **5b** was unsuccessful.

Given the previously reported coordinating studies of unsubstituted cyclam [16,19,29], the prepared new tetraamine ligands **5a**,**b** and **6a**,**b** (Scheme 2) were promising candidates for Au(III) coordination. Both ligands **5a** and **6a** readily coordinated with AuCl_3_ in methanol and gave moderate to excellent yields of tetracoordinated **5a**-Au(III) and **6a**-Au(III) *N,N,N,N*-complexes with alternating five- and six-membered chelate rings (50% and 96%, respectively, Scheme 3).

**Figure 1:**
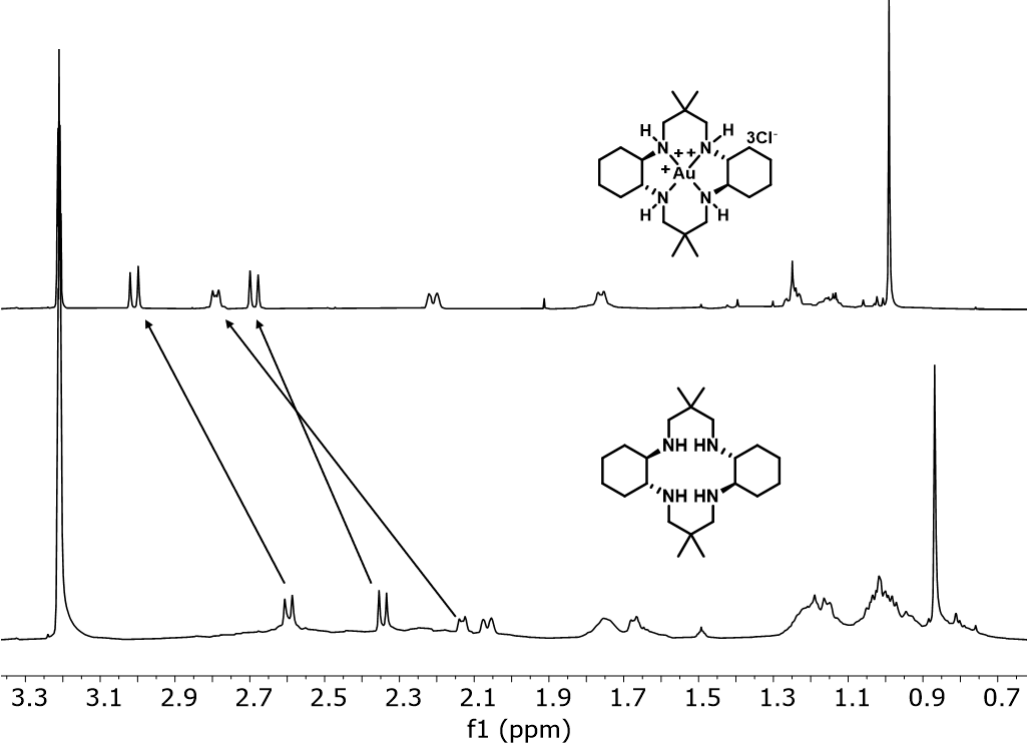
^1^H NMR study of the formation of complex **6a**-Au(III) by AuCl_3_ coordination to ligand **6a**.

Monitoring the formation of complex **5a**-Au(III), using ^1^H NMR, and ^1^H,^15^N-HMBC, clearly indicated a tetra-nitrogen-coordinated complex. This was evidenced by changes in NMR shift values, Δδ^15^N_coord_ = δ^15^N_complex_ – δ^15^N_ligand_, by coordination. The observed Δδ^15^N_coord_ values were in the range of 16.3–32.0 ppm for both the primary and secondary amine nitrogens, indicating a characteristic deshielding effect upon the Au(III) coordination [43,45–46], Likewise, Δδ^1^H_coord_ 0.3–0.5 ppm for all the neighboring N–C*H* and N–C*H*_2_ protons indicated ligand tetra-coordination to Au(III), as well. Upon coordination of ligand **5a**, four different ^15^N NMR values for the nitrogens were observed. This might be explained by the nitrogens becoming non-equivalent when coordinated to Au(III), as a result of the chiral centers in the ligand. The structure of **5a**-Au(III) was not confirmed, due to the lack of a suitable crystal for X-ray analysis, hence, only a proposed structure for **5a**-Au(III) is given (Scheme 3). Comparable effects for ligand **6a**, Δδ^1^H_coord_ 0.3–0.6 ppm, were also observed for the corresponding N–C*H* and N–C*H*_2_ neighboring protons by formation of complex **6a**-Au(III) (Figure 1).

Further on, cyclam **6b** readily coordinated to AuCl_3_ in a mixture of acetonitrile and dichloromethane, to obtain a sufficient solubility of cyclam **6b**, allowing formation of **6b**-Au(III) in 64% yield (Scheme 3). The corresponding Δδ^1^H_coord_ values of **6b**-Au(III) were similar to those discussed for **6a**-Au(III). Surprisingly, tetraamine **5b** did not behave in a similar way as the other ligands, instead giving a complex mixture, as judged by ^1^H NMR, when attempted coordinated to Au(III). Changing the source of Au(III) or the solvents methanol, acetonitrile and dichloromethane did not improve the outcome. Both purification and characterization of the Au(III) complexes were challenging as a result of low stability, and HRMS or elemental analysis could not be obtained, due to sample decomposition. Attempts to obtain crystals for X-ray analysis by slow diffusion of *n*-pentane into a DCM solution of the complexes were unsuccessful.

### Catalytic activity

For evaluation of the catalytic ability of the new Au(III) complexes, alkyne carboalkoxylation [47–48] and cyclopropanation of styrene with propargyl ester [49–52] (Table 1) were selected as test reactions. These reactions have previously been studied with different gold(I) and gold(III) catalysts and a variety of substrates, thus providing a solid background for comparison. A large difference in the catalytic activity was observed for cyclam–gold complex **6a**-Au(III) versus the open cyclam analogues **5a**-Au(III). Complex **5a**-Au(III) afforded a full conversion in the alkyne carboalkoxylation in 5.5 hours, compared to in 24 hours for complex **6a**-Au(III) (Table 1, entries 1 and 2). The same trend was observed for Au(III) catalysis of the cyclopropanation reaction, where complex **5a**-Au(III) and **6a**-Au(III) gave full conversion in 1 hour and 12 hours, respectively (Table 1, entries 4 and 5). The cyclopropyl product **11** was obtained in >90% yield and high *cis* diastereoselectivity (up to 74% de), similar to our previous studies [51], which showed that JohnPhos-Au(I) and pyr-menthol-Au(III) complexes provided high amounts of the initially formed *cis* diastereomer in this model reaction. In contrast, some BOX-Au(III) complexes have the additional ability to rapidly transform the initially formed *cis* product into the isomerized *trans* product. Thus, the proper choice of the gold catalyst allows highly stereoselective formation of either *cis* or *trans* cyclopropanation products and facilitates the isolation of pure isomers.

**Table 1:** The catalytic activity of Au(III) complexes evaluated in a) alkyne carboalkoxylation and b) cyclopropanation of styrene with propargyl ester.

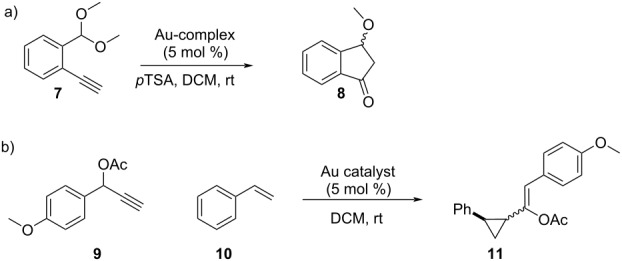			

Entry	Complex	Reaction time	Yield product

a) Carboalkoxylation of alkyne			**8**

1	**5a**-Au(III)	5.5 h	62%
2	**6a**-Au(III)	24 h	80%
3	**6b**-Au(III)	15 min	13%

b) Cyclopropanation			**11** (*cis*/*trans*)

4	**5a**-Au(III)	1 h	90% (77:23)
5	**6a**-Au(III)	12 h	97% (87:13)
6	**6b**-Au(III)	15 min	58% (12:88)
7	AuCl_3_	5 min	80% (13:87)

Despite the chiral nature of these ligands, no enantioselectivity was observed in the test reactions. Evaluation of complex **6b**-Au(III) in both reactions, revealed a large difference in catalytic activity and complex stability between the structurally similar **6a**-Au(III) and **6b**-Au(III) cyclam complexes, with a cyclohexyl and a diphenyl-C_2_ bridge between the nitrogens, respectively. In both test reactions, an immediate color change into dark red/brown took place after addition of complex **6b**-Au(III), indicating a low stabilization of the coordinated diphenyl ligand and a fast release of Au. The de-coordination resulted in full conversion within 15 min in both reactions (Table 1, entries 3 and 6), compared to 24 and 12 hours for complex **6a**-Au(III) (Table 1, entries 2 and 5), where the ligand seems to stabilize and deactivate the Au(III) during the reaction. Attempts to improve the **6b**-Au(III) complex stability by anion exchange with less coordinating anions failed, as addition of different standard silver salts resulted in decomposition of the Au(III) complex. Consequently, the counter-anion exchange method was not possible.

Since the ligand **6b** seems to de-coordinate, resulting in the cyclam Au(III) complex not being the active catalyst, and the presence of chloride anions, the activity of the **6b**-Au(III) precatalyst was compared to AuCl_3_. AuCl_3_ showed slightly faster conversion into the product, 5 min vs 15 min, however, a comparable *cis*/*trans* ratio was obtained. The reduced reaction time indicates that **6b**-Au(III) indeed is an precatalyst that needs some activation time before catalyzing the reaction.

Although the decomposition of complex **6b**-Au(III) resulted in the rapid conversion into products, it is undesirable, as the impact of the ligand on the reaction selectivity is lost. This different stability, caused by small differences in the design of the two cyclam ligands, is in accordance with the unsuccessful Au(III) coordination of the diphenyl-C_2_-bridged **5b** ligand, in contrast to the readily coordinating cyclohexyl-bridged **5a** tetraamine, as discussed above (Scheme 3).

## Conclusion

A new stepwise procedure was developed for improved preparation of chiral cyclam derivatives **5a**,**b** and **6a**,**b** from chiral mono-Boc-1,2-diamines and (dialkyl)malonyl dichloride. The four-step approach included ring closure of the initial open diamide–diamine intermediates **1** with a second malonyl unit, affording the cyclam tetraamides **2**. The target cyclam derivatives **5** and **6** were obtained by optimized LiAlH_4_ reduction by AlCl_3_ activation of the polyamides **1** and **2**.

Successful Au(III) coordination of the open tetraamine ligand **5a** and the new cyclam derivatives **6a**,**b** gave the corresponding tetracoordinated *N,N,N,N*-Au(III) cyclam **5a** and **6a**,**b** complexes (50–96%) with alternating five- and six-membered chelate rings. Verification of cyclam tetraamino-coordination was obtained by ^1^H,^15^N-HMBC NMR. The polyamides (**1**, **2**) failed to undergo Au(III) coordination, which confirmed the previously observed resistance of amides to coordinate to Au(III).

The catalytic ability of the new Au(III) complexes were screened in selected test reactions. A high catalytic ability was shown for novel *N,N,N,N*-Au(III) complexes **5a** and **6a** in alkyne carboalkoxylation and propargyl ester cyclopropanation (full conversion in 1–24 h, 62–97% product yields). No enantioselectivity was observed in the test reactions.

The activity and stability of the Au(III) complexes were strongly depending on the structure of the tetraamine ligands, demonstrating the importance of ligand design. Hence, the present study on cyclam based Au(III) complexes represents the first study on such chiral cyclam metal complexes and contributes to a better knowledge of the tetraamine ligand preparation and Au(III) coordination, as well as an increased understanding of Au(III) ligand design for optimal reaction outcomes.

## Supporting Information

File 1 Experimental procedures and NMR data for new ligands and gold(III) complexes, as well as a method for testing of catalytic activity.
