# Supplementary material for: Au(III) complexes with tetradentate-cyclam-based ligands
Source: Beilstein J Org Chem. 2021 Jan 19;17:186–92. doi: 10.3762/bjoc.17.18 (PMC7849238; doi:10.3762/bjoc.17.18)
Supplement: File 1 — Experimental procedures and NMR data for new ligands and gold(III) complexes, as well as a method for testing of catalytic activity. [file Beilstein_J_Org_Chem-17-186-s001.pdf]

BEILSTEIN JOURNAL OF ORGANIC CHEMISTRY

Supporting Information

for

Au(III) complexes with tetradentate-cyclam-based ligands

Ann Christin Reiersølmoen, Thomas N. Solvi and Anne Fiksdahl

Beilstein J. Org. Chem. **2021,** *17,* 186–192. doi:10.3762/bjoc.17.18

Experimental procedures and NMR data for new ligands and gold(III) complexes, as well as a method for testing of catalytic activity

License and Terms: This is a supporting information file under the terms of the Creative Commons Attribution License (https://creativecommons.org/licenses/by/4.0). Please note that the reuse, redistribution and reproduction in particular requires that the author(s) and source are credited and that individual graphics may be subject to special legal provisions.
The license is subject to the *Beilstein Journal of Organic Chemistry* terms and conditions: (https://www.beilstein-journals.org/bjoc/terms)

Table of contents

1. Experimental procedures S2
1.1 General Information: S2
1.2 Synthesis and characterization of ligands S2
1.3 Synthesis and characterization of gold(III) complexes S4
1.4 Synthesis and characterization of ligands unsuccessful for gold(III) coordination S4
1.5 General procedure for testing of catalytic activity. S5
2. Spectroscopic data S6
3. References S25

1. Experimental procedures

1.1 General:

Commercial grade reagents were used as received. Dry solvents were collected from a solvent-purification system. All reactions were monitored by thin-layer chromatography (TLC) using silica gel 60 F254 (0.25-mm thickness) or by ^{1}H NMR. Flash chromatography was carried out using silica gel 60 (0.040–0.063 mm). High Throughput Flash Purification (HPFP) was performed on pre-packed cartridges. ^{1}H, ^{13}C NMR, ^{1}H, ^{15}N HMBC NMR spectra were recorded in $CDCl_3$, CD_2Cl_2, CD_3CN or MeOD using a 400 or a 600 MHz Bruker spectrometer. ^{1}H and ^{13}C chemical shifts are reported in ppm (δ), using the residual solvent signal as internal standard. Accurate mass determination in either positive or negative mode was performed with a "Synapt G2-S" Q-TOF instrument from Waters. Samples were ionised with an ASAP probe, and no chromatographic separation was used before the mass analysis. 1-(Dimethoxymethyl)-2-ethynylbenzene, (**8**),[1] and 1-(4-methoxyphenyl)prop-2-yn-1-yl acetate (**11**) [2] were synthesized according to previously reported method.

1.2 Synthesis and characterization of ligands

*N*1,*N*3-bis((1*R*,2*R*)-2-Aminocyclohexyl)-2,2-dimethylmalonamide (**1a**)

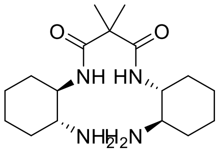

tert-Butyl((1*R*,2*R*)-2-aminocyclohexyl)carbamate (112 mg, 0.523 mmol) and triethylamine (73 µL, 0.523 mmol) were dissolved in dry THF (5 mL) under a nitrogen atmosphere. 2,2-Diethylmalonyl dichloride (35 µL, 0.261 mmol) was added to the reaction mixture and stirred for 2 hours. Small amounts of water were added before THF was removed under reduced pressure. The product mixture was dissolved in DCM (20 mL) and extracted with water (20 mL). The water phase was extracted with DCM (3 x 20 mL) and the combined organic phases were dried over Na_2SO_4, filtered and removed under reduced pressure. The crude product was directly boc-deprotected by addition of HCl (conc., 1 mL) in methanol (2 mL). The reaction mixture was stirred over night before aq. NaOH (1M) was added until the reaction mixture was basic. The water phase was extracted with DCM (4 x 20 mL) and the combined organic phases were dried over anhydrous Na_2SO_4 and filtered. No purification was needed. Dying gave the product as a pale powder, 65 mg (77 %, 0.201 mmol), mp.184.6 – 186.0 °C. ^{1}H NMR (600 MHz, MeOD) δ = 3.59 – 3.43 (m, 2H), 2.51 (ddd, *J*=11.3, 10.1, 4.1, 2H), 1.95 (ddt, *J*=12.5, 4.8, 2.3, 2H), 1.85 – 1.77 (m, 2H), 1.74 – 1.71 (m, 4H), 1.43 (s, 6H), 1.37 – 1.26 (m, 6H), 1.23 – 1.15 (m, 2H); ^{13}C NMR (151 MHz, MeOD) δ = 176.6, 57.1, 55.7, 52.1, 36.0, 32.9, 26.4, 26.2, 24.2; ^{15}N NMR (61 MHz, MeOD) δ = -254.6, -348.2. HRMS (APCI/ASAP, m/z): found 325.2603 (calcd. $C_{17}H_{33}N_4O_2$, 325.2604, $[M+H]^+$.

(4a*R*,9a*R*,13a*R*,18a*R*)-7,7,16,16-Tetramethylhexadecahydrodibenzo[*b*,*i*][1,4,8,11]tetraazacyclotetradecine-6,8,15,17(7*H*,16*H*)-tetraone (**2a**)

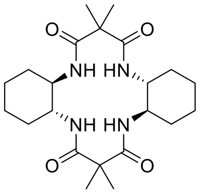

*N*1,*N*3-bis((1*R*,2*R*)-2-aminocyclohexyl)-2,2-dimethylmalonamide (80 mg, 247 mmol), **1a**, was dissolved in dry THF (4 mL) and added triethylamine (69 µL, 0.493 mmol), followed by addition of 2,2-diethylmalonyl dichloride (33 µL, 0.247 mmol). The reaction mixture was stirred for two hours before small amount of water was added. The reaction mixture was dried before extraction using DCM (3 × 20 mL) and water (20 mL). The combined organic phases were dried over Na_2SO_4, filtered and removed under reduced pressure to give the product as a pale powder, 83 mg (80%, 0.247 mmol). ^{1}H NMR (600 MHz, $CDCl_3$) δ = 6.84 (d, *J*=7.6, 4H), 3.60 (ddd, *J*=8.4, 6.2, 3.4, 4H), 1.96 (dt, *J*=12.8, 2.6, 4H), 1.76 (dt, *J*=6.8, 2.3, 4H), 1.39 (s, 12H), 1.34 – 1.19 (m, 8H); HRMS (APCI/ASAP, m/z): found 421.2816 (calcd. $C_{22}H_{37}N_4O_4$, 421.2815, $[M+H]^+$. The spectroscopic data corresponds to that previously reported.[3]

(1*R*,1'*R*,2*R*,2'*R*)-*N*1,*N*1'-(2,2-Dimethylpropane-1,3-diyl)bis(cyclohexane-1,2-diamine) (**5a**)

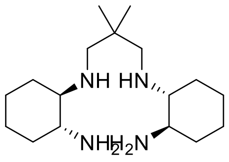

*N*1,*N*3-bis((1*R*,2*R*)-2-aminocyclohexyl)-2,2-dimethylmalonamide (262 mg, 0.946 mmol), **1a**, and $AlCl_3$ (262 mg, 1.965 mmol) were dried under vacuum, before they were dissolved in dry THF (8 mL). $LiAlH_4$ (1.5M, 5 mL, 7.570 mmol) was subsequently added at room temperature, before the reaction mixture was refluxed. The reaction mixture was stirred overnight, before it was quenched by addition of water. The water phase was extracted by DCM (10 × 20 mL). The combined organic phases were stirred with a sat. NaOH solution (50 mL) for an hour, before the two phases were separated. The organic phase was dried over anhydrous Na_2SO_4, filtered and removed under reduced pressure. This yielded the target product **5a** as a light yellow oil, 248 mg (88%, 0.946 mmol). ^{1}H NMR (600 MHz, MeOD) δ = 2.66 (d, *J* = 11.6 Hz, 2H), 2.37 (ddd, *J*=11.1, 9.5, 4.1, 2H), 2.26 (d, *J* = 11.6 Hz, 2H), 2.13 – 1.97 (m, 4H), 1.92 – 1.86 (m, 2H), 1.78 – 1.65 (m, 4H), 1.34 – 1.21 (m, 4H), 1.16 (tdd, *J*=12.8, 11.2, 3.7, 2H), 1.00 (tdd, *J*=12.5, 10.8, 3.6, 2H), 0.93 (s, 6H); ^{13}C NMR (151 MHz, MeOD) δ = 66.0, 57.0, 56.0, 36.2, 35.8, 32.2, 26.6, 26.3, 25.1; ^{15}N NMR (61 MHz, MeOD) δ = -341.7, -349.0. HRMS (APCI/ASAP, m/z): found 297.3018 (calcd. $C_{17}H_{37}N_4$, 297.3024, $[M+H]^+$.

(4a*R*,9a*R*,13a*R*,18a*R*)-7,7,16,16-Tetramethyldocosahydrodibenzo[b,i][1,4,8,11]tetraazacyclotetradecine, **6a**

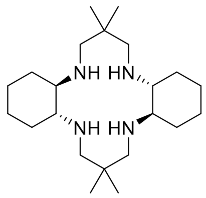

Cyclam **6a** was prepared according to literature procedure. The spectroscopic data corresponds to those previously reported.[3]

tert-Butyl ((1*R*,2*R*)-2-amino-1,2-diphenylethyl)carbamate, **B**-boc

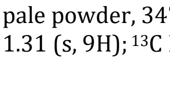

To a stirred solution of (1R,2R)-1,2-diphenylethane-1,2-diamine (365 mg, 1.72 mmol) in dry DCM (50 mL), di-*tert*-butyl decarbonate (340 mg, 1.56 mmol) dissolved in dry DCM (25 mL) was added dropwise over the course of 15 mins. After stirring for 2 days, $NaHCO_3$ (250 mg) was added and the mixture was concentrated under reduced pressure. The solution was washed with aqueous NaOH (1 M, 2 × 15 mL) and dried over Na_2SO_4, before the solvent was removed in vacuo. Purification by flash column chromatography (EtOAc) gave pure product as a pale powder, 347 mg (71%, 1.11 mmol). ^{1}H NMR (400 MHz, $CDCl_3$) δ= 7.37 – 7.21 (m, 10H), 5.87 (m, 1H), 4.85 (s, 1H), 4.32 (s, 1H), 1.31 (s, 9H); ^{13}C NMR (101 MHz, $CDCl_3$) δ= 155.7, 142.3, 141.1, 128.5 (2C), 128.3 (2C), 127.4, 127.2, 126.8 (2C), 126.5 (2C), 79.2,

60.0 (2C), 28.3 (3C); HRMS (APCI/ASAP, m/z): found 335.1735 (calcd. $C_{19}H_{24}N_2O_2Na$, 335.1733, $[M+Na]^+$). The spectroscopic data is in accordance with that reported previously.[4]

Di-*tert*-butyl((1*R*,1'*R*,2*R*,2'*R*)-((2,2-dimethylmalonyl)bis(azanediyl))bis(1,2-diphenylethane-2,1-diyl))dicarbamate (**1b'**)

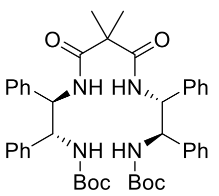

Tert-butyl ((1*R*,2*R*)-2-amino-1,2-diphenylethyl)carbamate, **B**-boc (106 mg, 0.34 mmol) and NEt_3 (190 μL, 1.36 mmol) were dissolved in dry THF (1 mL) and stirred for 5 minutes, after which dimethylmalonyl chloride (23 μL, 0.17 mmol) was added dropwise. More THF was added (4 mL) and the solution stirred at rt overnight. H_2O (5 mL) was added and the solution filtered, leaving the product **1b'** as white flakes, 102 mg (82%, 0.279 mmol). ^1H NMR (600 MHz, d_6-DMSO) δ= 7.76 (d, *J* = 9.2, 2H), 7.66 (d, *J* = 9.5, 2H), 7.26 (d, *J* = 7.4, 4H), 7.22 (t, *J* = 7.4, 4H), 7.11-7.20 (m, 12H), 5.39 (dd, *J* = 8.8, 6.0, 2H), 5.10 (dd, *J* = 9.4, 6.0, 2H), 1.22 (s, 18H), 0.91 (s, 6H); ^{13}C NMR (150 MHz, d_6-DMSO) δ= 172.2 (2C), 155.2 (2C), 140.8 (2C), 140.2 (2C), 127.7 (6C), 127.6 (4C), 126.9 (6C), 126.6 (2C), 126.4 (2C), 77.9 (2C), 57.8 (2C), 56.9 (2C), 49.6, 28.1 (6C), 23.5 (2C); HRMS (ESI, m/z): found 742.3785 (calcd. $C_{43}H_{52}N_4O_6Na$, 743.3782, $[M+Na]^+$).

*N*¹,*N*³-Bis((1*R*,2*R*)-2-amino-1,2-diphenylethyl)-2,2-dimethylmalonamide (**1b**)

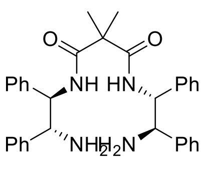

Chiral dicarbamate **1b'** (102 mg, 0.140 mmol) was dissolved in TFA (1 mL) at 0 °C, and the solution was stirred for 20 min. Aqueous NaOH (1 M) was added dropwise until ~pH 10, followed by addition of DCM (10 mL). The phases were separated and the aqueous phase was extracted with more DCM (3 × 10 mL). The combined organic phases were washed with H_2O (10 mL), dried over Na_2SO_4, and concentrated in vacuo, yielding pure product **1b**, 71 mg (95 %, 0.133 mmol), as a pale solid. ^1H NMR (600 MHz, $CDCl_3$) δ= 7.99 (d, *J* = 8.0, 2H), 7.36 (d, *J* = 7.4, 4H), 7.29 (t, *J* = 7.5, 4H), 7.21-7.25 (m, 8H), 7.15-7.18 (m, 4H), 5.10 (dd, *J* = 8.1, 3.3, 2H), 4.36 (d, *J* = 2.8, 2H), 1.29 (s, 6H), 1.22 (bs, 4H); ^{13}C NMR (150 MHz, $CDCl_3$) δ= 173.1 (2C), 142.2 (2C), 140.5 (2C), 128.7 (4C), 128.5 (4C), 127.6 (2C), 127.3 (2C), 126.7 (4C), 126.4 (4C), 59.7 (2C), 58.9 (2C), 49.6, 24.1 (2C); HRMS (APCI/ASAP, m/z): found 521.2917 (calcd. $C_{33}H_{37}N_4O_2$, 521.2926, $[M+H]^+$).

(1*R*,1'*R*,2*R*,2'*R*)-N¹,N¹'-(2,2-Dimethylpropane-1,3-diyl)bis(1,2-diphenylethane-1,2-diamine) (**5b**)

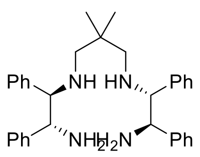

Compound **1b** (71 mg, 0.136 mmol) and $AlCl_3$ (73 mg, 0.54 mmol) were dissolved in dry THF (5 mL) and cooled to 0 °C. After stirring for 15 mins, $LiAlH_4$ (1 M, 2.7 mL, 2.7 mmol) was added and the solution was stirred overnight. H_2O (20 mL) and NaOH were added until ~pH 14. The solution was filtered and extracted with DCM (3 × 10 mL), dried over Na_2SO_4 and concentrated in vacuo yielding 19.5 mg (29 %, 0.040 mmol) of **5b** as a pale powder. ^1H NMR (400 MHz, $CDCl_3$) δ= 7.05-7.24 (m, 20H), 3.93 (d, *J* = 7.1, 2H), 3.59 (d, *J* = 7.1, 2H), 2.21 (d, *J* = 11.4, 2H), 2.14 (d, *J* = 11.4, 2H), 1.79 (bs, 6H), 0.79 (s, 6H); ^{13}C NMR (100 MHz, $CDCl_3$) δ= 143.9 (2C), 142.2 (2C), 128.13 (4C), 128.06 (4C), 127.9 (4C), 127.3 (4C), 126.94 (2C), 126.90 (2C), 70.8 (2C), 62.2 (2C), 57.2 (2C), 35.3, 24.8 (2C); HRMS (APCI/ASAP, m/z): found 493.3337 (calcd. $C_{33}H_{41}N_4$, 493.3337, $[M+H]^+$).

(2*R*,3*R*,9*R*,10*R*)-6,6,13,13-Tetramethyl-2,3,9,10-tetraphenyl-1,4,8,11-tetraazacyclotetradecane-5,7,12,14-tetraone (**2b**)

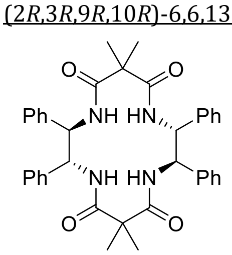

(1*R*,2*R*)-(+)-1,2-Diphenylethylenediamine (265 mg, 1.248 mmol) and triethylamine (174 µL, 1.248 mmol mmol) were dissolved in dry THF (15 mL) under a nitrogen atmosphere. 2,2-Dimethylmalonyl dichloride (165 µL, 1.248 mmol) was added dropwise to the reaction mixture and stirred overnight. Small amounts of water were added before THF was removed under reduced pressure. The product mixture was dissolved in DCM (30 mL) and extracted with water (30 mL). The water phase was extracted with DCM (3 × 20 mL) and the combined organic phases were dried over Na_2SO_4, filtered and removed under reduced pressure. The crude product was purified by silica gel column chromatography (EtOAc, $R_{f,\,\mathbf{1}}$ = 0.56, $R_{f,\,\mathbf{2}}$ = 0.63). Drying gave 250 mg (65%, 0.405 mmol) of the desired product **2b** as a white powder, mp 251.4 – 253.2 °C, (and 43 mg of byproduct **2b-trimer)**. ^1H NMR (600 MHz, $CDCl_3$) δ = 7.74 (dd, *J*=5.6, 2.8, 4H), 7.23 – 7.19 (m, 12H), 7.11 – 7.10 (m, 8H), 5.25 (dd, *J*=5.7, 2.7, 4H), 1.43 (s, 12H); ^{13}C NMR ($CDCl_3$, 100 MHz) δ= 174.2 (4C), 138.1 (4C), 128.8 (8C), 128.1 (4C), 127.6 (8C), 60.1 (4C), 49.5 (2C), 23.5 (4C); HRMS (APCI/ASAP, m/z): found 617.3127 (calcd. $C_{38}H_{41}N_4O_4$, 617.3128, $[M+H]^+$).

(2*R*,3*R*,9*R*,10*R*)-6,6,13,13-Tetramethyl-2,3,9,10-tetraphenyl-1,4,8,11-tetraazacyclotetradecane, (**6b**)

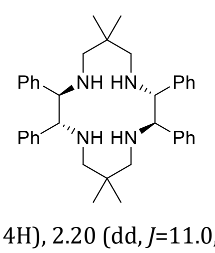

Compound **2b** (100 mg, 0.162 mmol) and $AlCl_3$ (90 mg, 0.675 mmol) were dissolved in dry THF (3 mL) under a nitrogen atmosphere, before $LiAlH_4$ (6 mL9, 1.5M in THF, 9.00 mmol) was added at room temperature. The reaction mixture was refluxed for two days, before the reaction was quenched by addition of water. The crude product was extracted with DCM (8 × 30 mL), before the combined organic phases were washed with sat. NaOH solution (50 mL). The two phases were separated and the organic phase was dried over anhydrous Na_2SO_4, filtered and dried under reduced pressure. The crude product was purified by precipitation from DCM with *n*-pentane, yielding the product as a pale powder, 59 mg (65%, 0.105 mmol). ^1H NMR (600 MHz, $CDCl_3$) δ = 7.17 (dd, *J*=8.3, 6.4, 8H), 7.15 – 7.07 (m, 12H), 3.57 (d, *J*=3.9, 4H), 2.90 (s, 4H), 2.43 (dd, *J*=11.1, 7.6, 4H), 2.20 (dd, *J*=11.0, 7.4, 4H), 0.73 (s, 12H); ^{13}C NMR (151 MHz, $CDCl_3$) δ = 142.4 (4C), 128.1 (8C), 127.9 (8C), 126.7 (4C), 70.3 (4C), 58.4 (4C), 34.5 (2C), 25.8 (4C); HRMS (APCI/ASAP, m/z): found 561.3960 (calcd. $C_{38}H_{49}N_4$, 561.3957, $[M+H]^+$).

1.3 Synthesis and characterization of gold(III) complexes

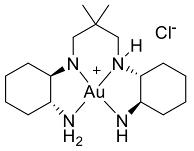

5a-Au(III):

(1*R*,2*R*)-*N*1-(3-(((1*R*,2*R*)-2-(l2-Azaneyl)cyclohexyl)amino)-2,2-dimethylpropyl)cyclohexane-1,2-diamine, **5a**, (15 mg, 0.049 mmol) was dissolved in methanol and added $KAuCl_4$ (19 mg, 0.049 mmol) dissolved in methanol. The complex was dissolved in dichloromethane and filtered to remove KI precipitate. Upon drying the complex, 13 mg (50%, 0.024 mmol) was obtained of the complex **5a**-Au(III) as a yellow solid. ^{1}H NMR (600 MHz, MeOD) δ: 4.03 – 3.79 (m, 1H), 3.57 – 3.49 (m, 1H), 3.47 – 3.39 (m, 3H), 3.31 – 3.11 (m, 1H), 2.92 (d, *J* = 13.1 Hz, 1H), 2.60 (d, *J* = 12.8 Hz, 1H), 2.58 – 2.42 (m, 1H), 2.39 – 2.14 (m, 3H), 1.95 – 1.70 (m, 5H), 1.74 – 1.55 (m, 3H), 1.43 – 1.29 (m, 4H), 1.27 (s, 3H), 1.18 (s, 3H); ^{13}C NMR (151 MHz, MeOD) δ = 73.6, 70.9, 64.5, 63.8, 59.7, 54.9, 38.5, 34.0, 33.9, 31.1, 30.7, 26.4, 25.9, 25.7, 25.0, 24.9, 22.5; ^{15}N NMR (61 MHz, MeOD) δ = -332.7, -329.3, -315.6, -309.7; HRMS; could not be detected.

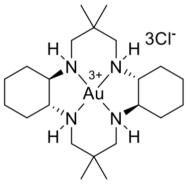

6a-Au(III):

(4a*R*,9a*R*,13a*R*,18a*R*)-7,7,16,16-Tetramethyldocosahydrodibenzo[b,i][1,4,8,11]tetraazacyclotetradecine, **6a**, (3 mg, 8,2 µmol) was dissolved in methanol and added $AuCl_3$ (3 mg, 8.2 µmol) dissolved in methanol. Upon drying the complex, 5 mg (96%, 8.2 µmol) was obtained of the complex **6a**-Au(III) as a yellow solid. ^{1}H NMR (600 MHz, MeOD) δ 3.11 (d, *J* = 13.1 Hz, 4H), 2.92 – 2.88 (m, 4H), 2.79 (d, *J* = 13.1 Hz, 4H), 2.39 – 2.24 (m, 4H), 1.93 – 1.79 (m, 4H), 1.40 – 1.29 (m, 4H), 1.29 – 1.21 (m, 4H), 1.09 (s, 12H); ^{13}C NMR (151 MHz, MeOD) δ = 62.4 (4C), 56.8 (4C), 33.7 (2C), 29.3 (4C), 25.4 (4C), 24.6 (4C); ^{15}N NMR (61 MHz, MeOD) δ = -338.4; HRMS; could not be detected.

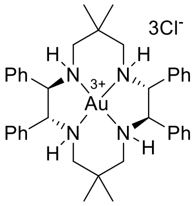

6b-Au(III):

(2*R*,3*R*,9*R*,10*R*)-6,6,13,13-Tetramethyl-2,3,9,10-tetraphenyl-1,4,8,11-tetraazacyclotetradecane, **6b**, (3 mg, 5.7 µmol) was dissolved in a mixture of acetonitrile (0.5 mL) and dichloromethane (0.5 mL), before adding $AuCl_3$ (2 mg, 5.7 µmol), dissolved in acetonitrile (1 mL). The coordinating mixture was stirred for 10 min before the solvent was removed under reduced pressure. After drying, the complex **6b**-Au(III) was obtained as a yellow solid, 2.9 mg (64%, 3.7 µmol). ^{1}H NMR (600 MHz, MeOD) δ = 7.25 (d, *J* = 6.9 Hz, 8H), 7.22 – 7.12 (m, 12H), 4.51 (s, 4H), 2.76 (d, *J* = 13.0 Hz, 4H), 2.66 (d, *J* = 13.8 Hz, 4H), 0.95 (s, 12H); ^{13}C NMR (151 MHz, MeOD) δ = 134.7 (4C), 130.3 (4C), 130.2 (16C), 69.1 (4C), 58.2 (4C), 34.1 (2C), 24.6 (4C); ^{15}N NMR (61 MHz, MeOD) δ = -333.5; HRMS; could not be detected.

1.4 Synthesis and characterization of ligands unsuccessful for gold(III) coordination

General Method: Amine (2 equiv) and triethylamine (2 equiv) were dissolved in dry THF under a nitrogen atmosphere. 2,2-Dimalonyl dichloride (1 equiv) was added dropwise to the reaction mixture and the mixture was stirred for 1–4 hours. Small amounts of water were added before THF was removed under reduced pressure. The product mixture was dissolved in DCM and extracted with water. The water phase was extracted with DCM and the combined organic phases were dried over Na_2SO_4, filtered and removed under reduced pressure. If purification was needed, the method is specified for each compound.

*N*1,*N*3-Bis((1*S*,2*S*)-2-(diphenylphosphanyl)cyclohexyl)-2,2-dimethylmalonamide (**1c**)

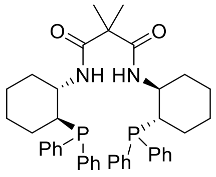

Product **1c** was synthesized according to General Method using (1*S*,2*S*)-2-(diphenylphosphaneyl)cyclohexan-1-amine (21 mg, 0.074 mmol), triethylamine (11 µL, 0.074 mmol) and 2,2-dimethylmalonyldichloride (5 µL, 0.037 mmol). The crude product was purified by silica gel column chromatography (EtOAc, R_f = 0.65). Drying gave 16 mg (65%, 0.024 mmol) of **1c** as a white powder. ^{1}H NMR (600 MHz, $CDCl_3$) δ = 8.72 (d, *J*=8.4, 2H), 7.86 – 7.76 (m, 4H), 7.75 – 7.68 (m, 4H), 7.59 (td, *J*=7.4, 1.5, 2H), 7.56 – 7.44 (m, 10H), 4.14 (dt, *J*=9.3, 4.6, 2H), 2.56 (dtd, *J*=13.6, 10.5, 3.1, 2H), 1.93 (dt, *J*=13.2, 4.1, 2H), 1.68 (d, *J*=9.8, 2H), 1.62 – 1.60 (m, 2H), 1.51 – 1.40 (m, 2H), 1.37 (s, 6H), 1.24 – 1.14 (m, 4H), 1.04 – 0.96 (m, 4H); ^{13}C NMR (151 MHz, $CDCl_3$) δ = 173.0 (2C), 133.0 (d, $^{1}J_{C-P}$ = 96.1 Hz, 2C), 132.5 (d, $^{3}J_{C-P}$ = 8.9 Hz, 4C), 131.9 (d, $^{4}J_{C-P}$ = 1.9 Hz, 2C), 131.8 (d, $^{4}J_{C-P}$ = 1.2 Hz, 2C), 131.3 (d, $^{3}J_{C-P}$ = 8.7 Hz, 4C), 130.0 (d, $^{1}J_{C-P}$ = 96.3 Hz, 2C), 128.7 (d, $^{2}J_{C-P}$ = 11.1 Hz, 4C), 128.3 (d, $^{2}J_{C-P}$ = 11.2 Hz, 4C), 50.4, 47.1 (d, $^{2}J_{C-P}$ = 3.9 Hz, 2C), 41.5 (d, $^{1}J_{C-P}$ = 69.2 Hz, 2C), 33.8 (d, $^{3}J_{C-P}$ = 8.8 Hz, 2C), 27.9 (2C), 25.2 (d, $^{2}J_{C-P}$ = 13.0 Hz, 2C), 24.9 (2C), 24.5 (2C); ^{31}P NMR (162 MHz, $CDCl_3$) δ = 37.2; HRMS (APCI/ASAP, m/z): found 695.3170 (calcd. $C_{41}H_{49}N_2O_4P_2$, 695.3168, $[M+H]^+$).

2,2-Dimethyl-*N1*-((*R*)-1-phenylethyl)-*N*3-((*S*)-1-phenylethyl)malonamide (**1d**)

Product **1d** was synthesized according to General Method starting with (*R*)-1-phenylethan-1-amine (75 µL, 0.590 mmol), triethylamine (82 µL, 0.590 mmol) and 2,2-dimethylmalonyldichloride (39 µL, 0.295 mmol). No purification was needed. Drying gave 91 mg (91%, 0.268 mmol) of **1d** as a white powder, mp 159.1 – 160.2 °C. ^{1}H NMR ($CDCl_3$, 400 MHz) δ = 7.35-7.20 (m, 10H), 6.80 (ap. d, *J* = 7.0, 2H), 5.05 (p, *J* = 7.0, 2H), 1.46 (d, *J* = 6.8, 6H), 1.45 (s, 6H); ^{13}C NMR ($CDCl_3$, 100 MHz) δ = 172.6 (2C), 143.0 (2C), 128.7 (4C), 127.3 (2C), 125.9 (4C), 40.24, 49.16 (2C), 23.9 (2C), 21.9 (2C); HRMS (APCI/ASAP, m/z): found 339.2068 (calcd. $C_{21}H_{27}N_2O_2$, 339.2073, $[M+H]^+$).

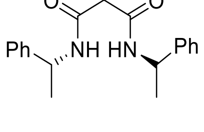

N^1-((*R*)-2-Hydroxy-1-phenylethyl)-N^3-((*S*)-2-hydroxy-1-phenylethyl)-2,2-dimethylmalonamide (**1e**)

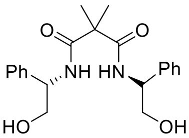

Product **1e**, previously reported in the literature,[5] was synthesized according to General Method using (*S*)-2-amino-2-phenylethan-1-ol (82 mg, 0.598 mmol), triethylamine (82 μL, 0.590 mmol) and 2,2-dimethylmalonyldichloride (39 μL, 0.295 mmol). No purification was needed. Drying gave 83 mg (76 %, 0.224 mmol) of **1e** as a white powder, mp 124.1 – 126.0 °C.^{1}H NMR (400 MHz, $CDCl_3$) δ = 7.40 – 7.18 (m, 10H), 7.12 (d, *J*=7.8, 2H), 5.12 (td, *J*=7.3, 3.9, 2H), 3.92 (dd, *J*=11.5, 4.0, 2H), 3.79 (dd, *J*=11.5, 7.0, 2H), 1.52 (s, 6H); ^{13}C NMR (101 MHz, $CDCl_3$) δ = 174.0 (2C), 138.4 (2C), 128.9 (4C), 127.9 (2C), 126.5 (4C), 66.3 (2C), 55.7 (2C), 49.9, 23.7 (2C); HRMS (APCI/ASAP, m/z): found 393.1797 (calcd. $C_{21}H_{26}N_2O_4Na$, 393.1790, $[M+Na]^+$).

N^1,N^3-Bis((1*R*,2*R*)-2-aminocyclohexyl)-2,2-diethylmalonamide (**3a**)

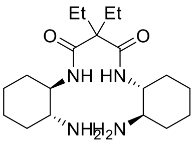

t-Butyl((1*R*,2*R*)-2-aminocyclohexyl)carbamate (113 mg, 0.527 mmol) and triethylamine (73 μL, 0.527 mmol) were dissolved in dry THF (5 mL) under a nitrogen atmosphere. 2,2-Diethylmalonyl dichloride (45 μL, 0.261 mmol) was added to the reaction mixture and stirred overnight. Small amounts of water were added before THF was removed under reduced pressure. The product mixture was dissolved in DCM (20 mL) and extracted with water (20 mL). The water phase was extracted with DCM (3 × 20 mL) and the combined organic phases were dried over Na_2SO_4, filtered and removed under reduced pressure. The crude product was directly boc-deprotected by addition of DCM (5 mL) and TFA (2 mL). The reaction mixture was stirred over night before aq. NaOH (1 M) was added until the reaction mixture was basic. The water phase was extracted with DCM (4 × 20 mL) and the combined organic phases were dried over anhydrous Na_2SO_4 and filtered. Drying under reduced pressure yielded 71 mg (66%, 0.201 mmol) of the unprotected product **3a** as a white powder. ^{1}H NMR ($CDCl_3$, 600 MHz) δ = 7.10 (d, *J* =8.2, 2H), 3.61-3.55 (m, 2H), 2.40 (td, *J* = 10.5, 6.4, 3.5, 2H), 1.96-1.94 (m, 4H), 1.91-1.87 (m, 4H), 1.73-1.71 (m, 4H), 1.36-1.31 (m, 2H), 1.30-1.24 (m, 2H), 1.22-1.18 (m, 2H), 1.17-1.14 (m, 2H), 0.87 (t, *J* = 7.1, 6H); ^{13}C NMR ($CDCl_3$, 150 MHz) δ: 173.3 (2C), 58.4, 55.8 (2C), 55.6 (2C), 36.2 (2C), 32.4 (2C), 29.2 (2C), 25.1 (2C), 25.1 (2C), 9.3 (2C); HRMS (APCI/ASAP, m/z): found 353.2912 (calcd. $C_{19}H_{37}N_4O_2$, 353.2917, $[M+H]^+$).

(4a*R*,9a*R*,13a*R*,18a*R*)-7,7,16,16-Tetraethylhexadecahydrodibenzo[*b*,*i*][1,4,8,11]tetraazacyclotetradecine-6,8,15,17(7*H*,16*H*)-tetraone (**4a**)

Product **3a** (24 mg, 0.068 mmol) was dissolved in dry THF (5 mL) and added triethylamine (20 μL, 0.136 mmol) under a nitrogen atmosphere. Diethylmalonyl dichloride (12 μL, 0.068 mmol) was added to the reaction mixture and stirred overnight. Small amounts of water were added before THF was removed under reduced pressure. The product mixture was dissolved in DCM (20 mL) and extracted with aq. NaOH (1M, 20 mL). The water phase was extracted with DCM (5 × 20 mL) and the combined organic phases were dried over Na_2SO_4, filtered and removed under reduced pressure. Drying gave 31 mg (95%, 0.065 mmol) of product **4a** as a white powder. ^{1}H NMR (600 MHz, $CDCl_3$) δ = 6.71 (d, *J*=6.1, 4H), 3.61 – 3.44 (m, 4H), 2.14 – 1.95 (m, 4H), 1.90 (qd, *J*=7.5, 2.2, 8H), 1.79 – 1.71 (m, 4H), 1.29 (tt, *J*=9.4, 2.4, 4H), 1.26 – 1.15 (m, 4H), 0.80 (t, *J*=7.5, 12H); ^{13}C NMR ($CDCl_3$, 150 MHz) δ = 172.9 (4C), 57.3 (2C), 54.4 (4C), 32.0 (4C), 24.6 (4C), 22.9 (4C), 8.1 (4C); HRMS (APCI/ASAP, m/z): found 477.3436 (calcd. $C_{26}H_{45}N_4O_4$, 477.3441, $[M+H]^+$).

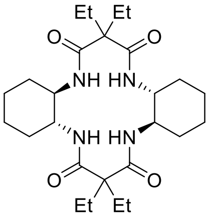

1.5 General procedure for testing of catalytic activity.

The catalytic activity of the different Au(III) complexes described in Section 1.3 were evaluated in two model reactions;
a) carboalkoxylation of alkyne and b) cyclopropanation of styrene with propargyl ester (Table 1a,b), as described below.

a) Carboalkoxylation of alkyne:[4]

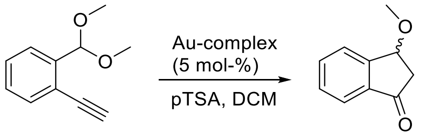

1-(Dimethoxymethyl)-2-ethynylbenzene (10 mg, 0.053 mmol) was dissolved in dichloromethane and added to the gold(III) catalyst (5 mol %). The reaction mixture was added *p*-toluenesulfonic acid (cat.) when TLC indicted full conversion of the starting material. The crude product was dried and purified by silica column chromatography (*n*-pentane:ethylacetate 10:1, R_f = 0.46). Reactivity data, yield and enantioselectivity for the different Au-catalyst is presented in the main text, Table 1a.

b) Cyclopropantion of styrene with propargyl ester:[5]

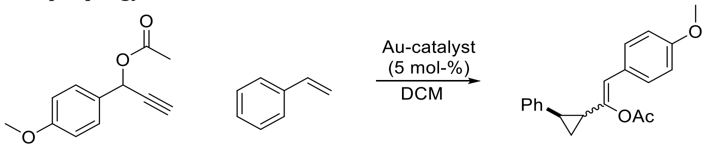

The propargyl ester (10 mg, 1 equiv.) and styrene (4 equiv) were dissolved in *d*-DCM (0.6 mL) and added the gold-catalyst (5 mol %) dissolved in *d*-DCM. The reaction progress was monitored by ^{1}H NMR or TLC, while the *cis*/*trans* ration were determined by ^{1}H NMR. The crude product was purified by silica column chromatography (*n*-pentane:ethylacetate 10:1, R_f = 0.36 (*cis*), 0.34 (*trans*)). Reactivity data, yield and enantioselectivity for the different Au-catalyst is presented in the main text, Table 1b.

2. Spectroscopic data

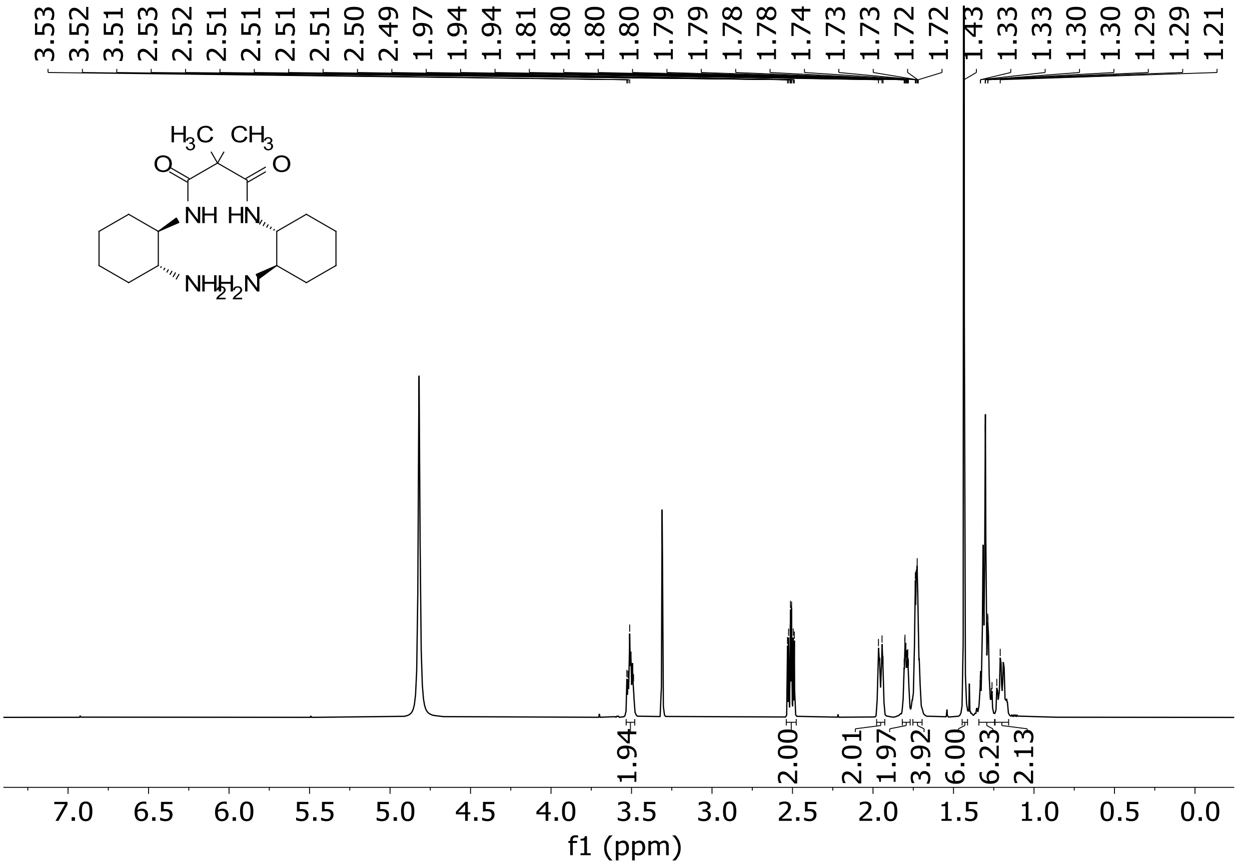

Figure S1. The ^{1}H NMR spectrum of **1a** acquired at 25 °C in CD_3OD at 600 MHz.

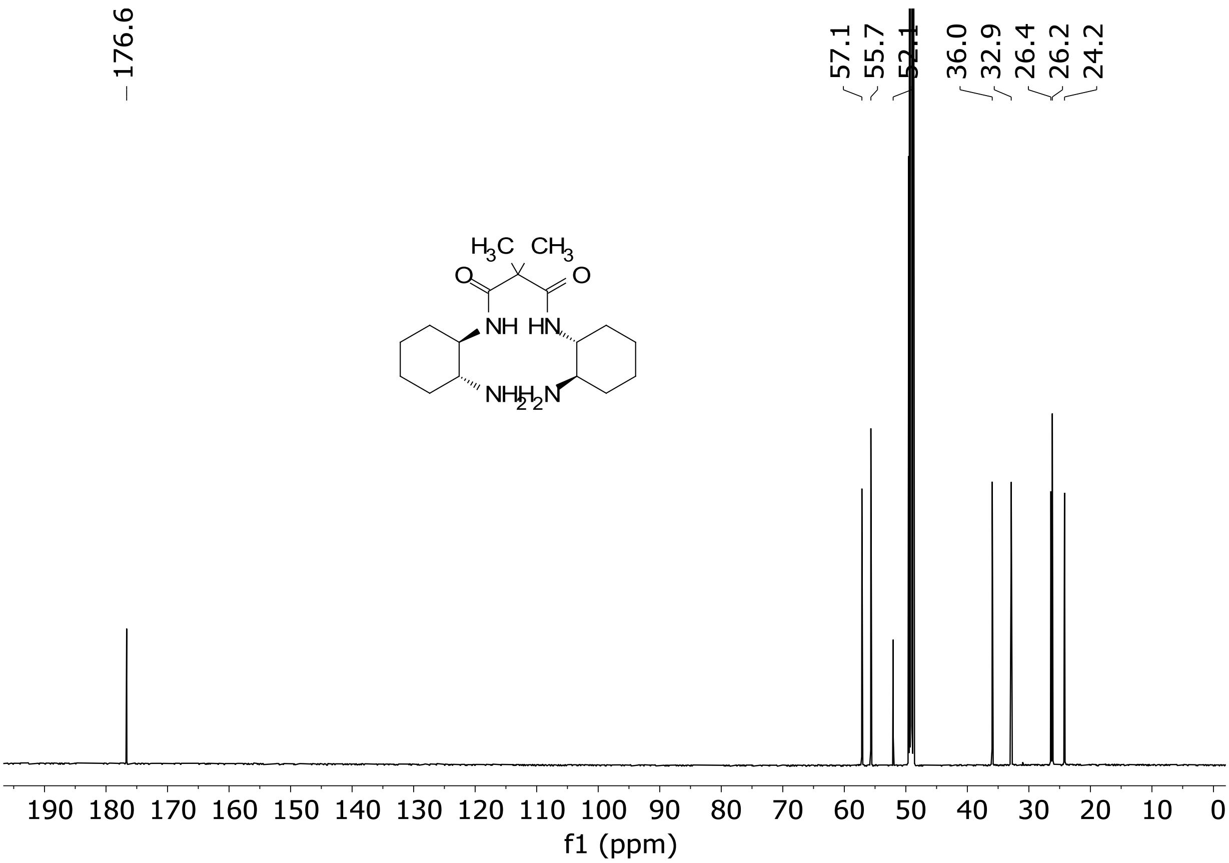

Figure S2. The ^{13}C NMR spectrum of **1a** acquired at 25 °C in CD_3OD at 151 MHz.

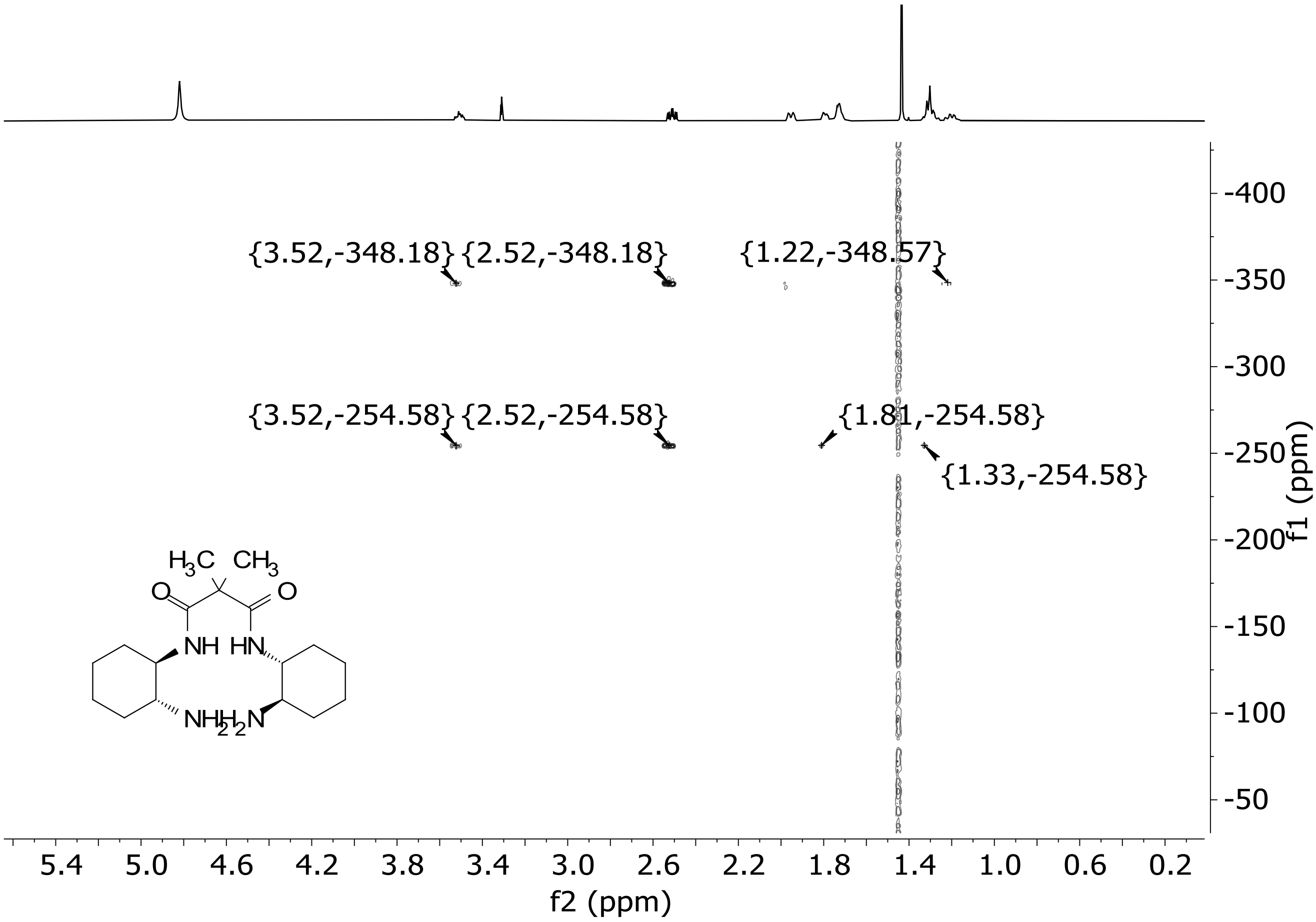

Figure S3. The ^{1}H, ^{15}N HMBC NMR spectrum of **1a** acquired at 25 °C in CD_3OD at 61 MHz.

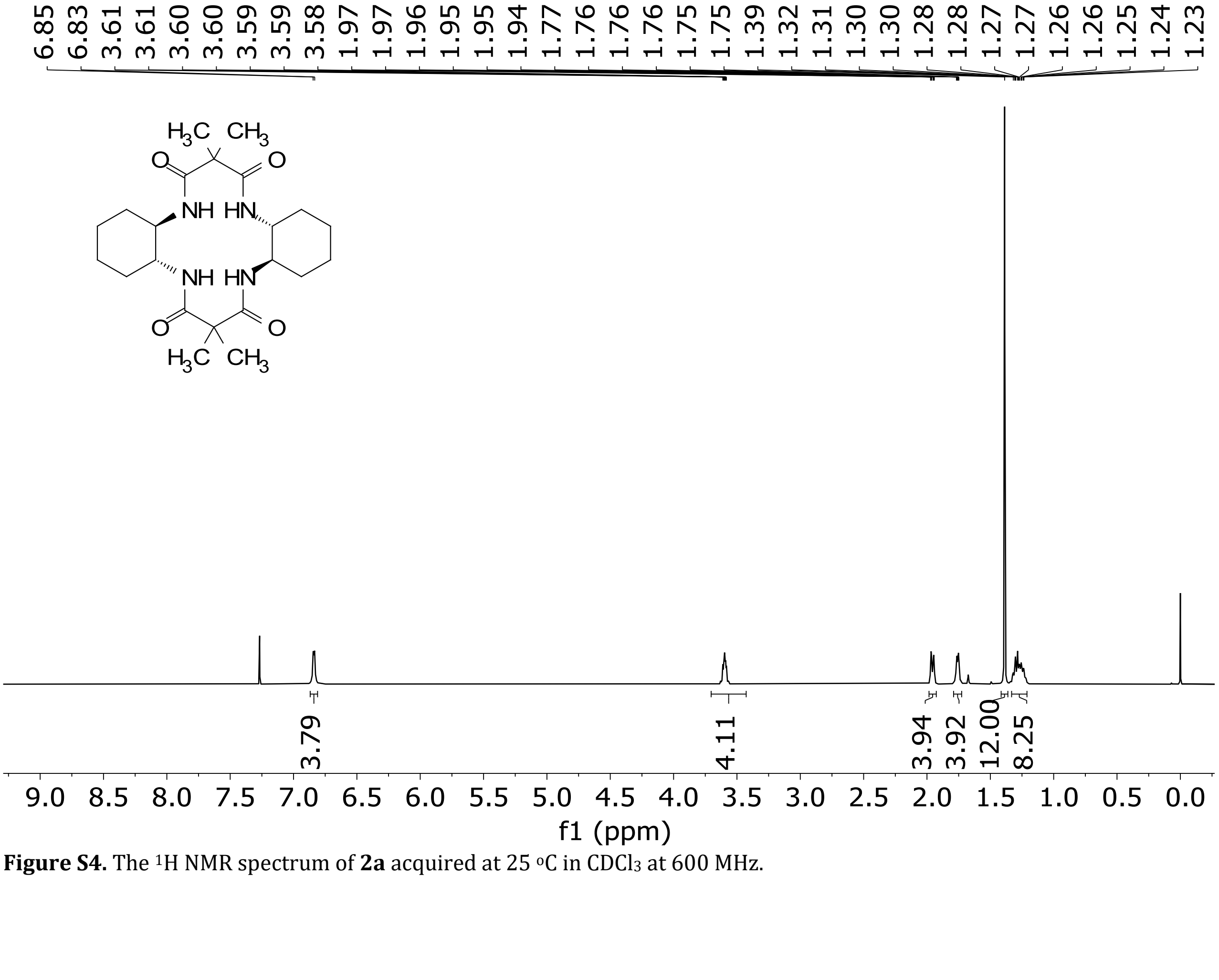

Figure S4. The ^{1}H NMR spectrum of **2a** acquired at 25 °C in $CDCl_3$ at 600 MHz.

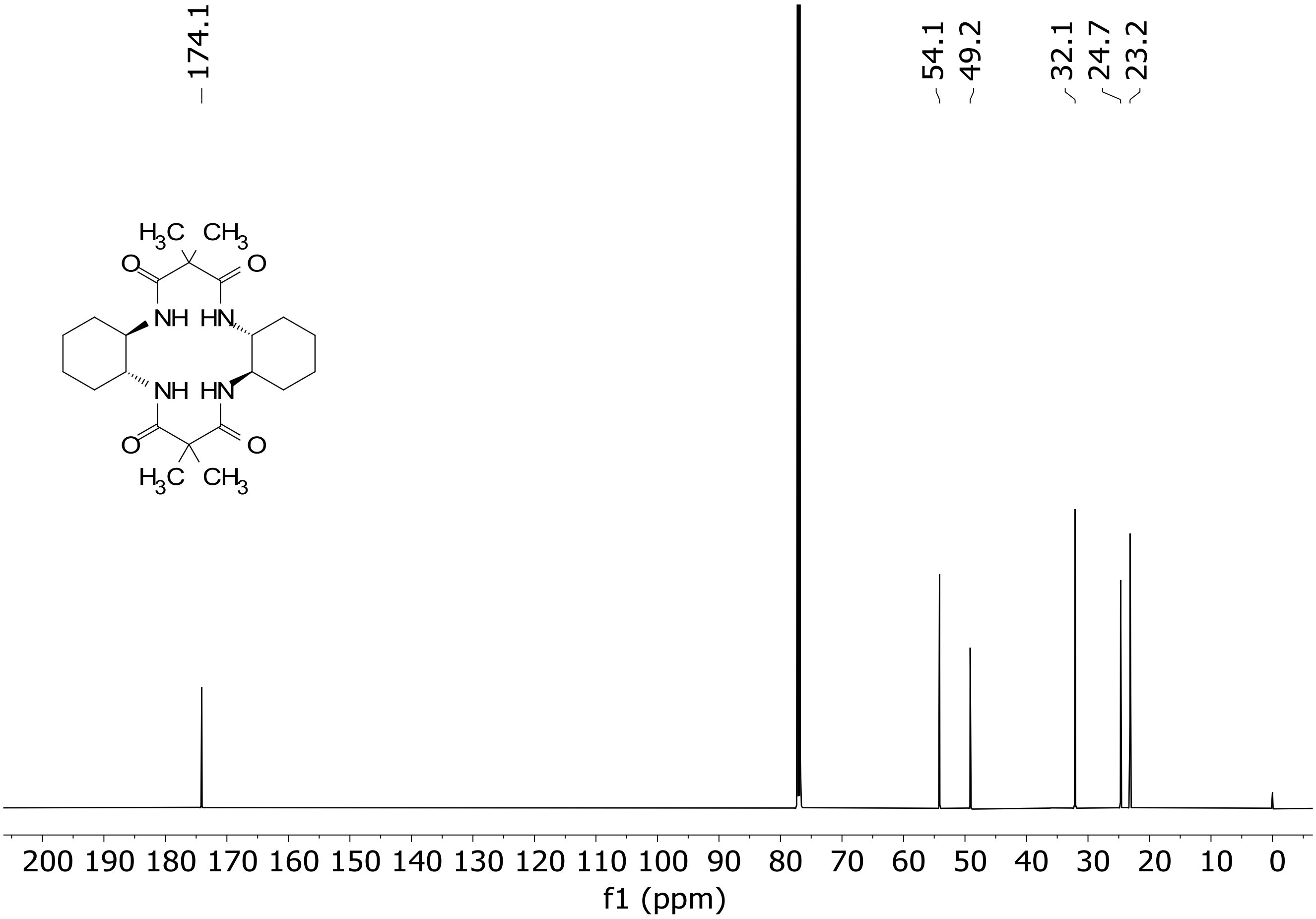

Figure S5. The ^{13}C NMR spectrum of **2a** acquired at 25 °C in $CDCl_3$ at 151 MHz

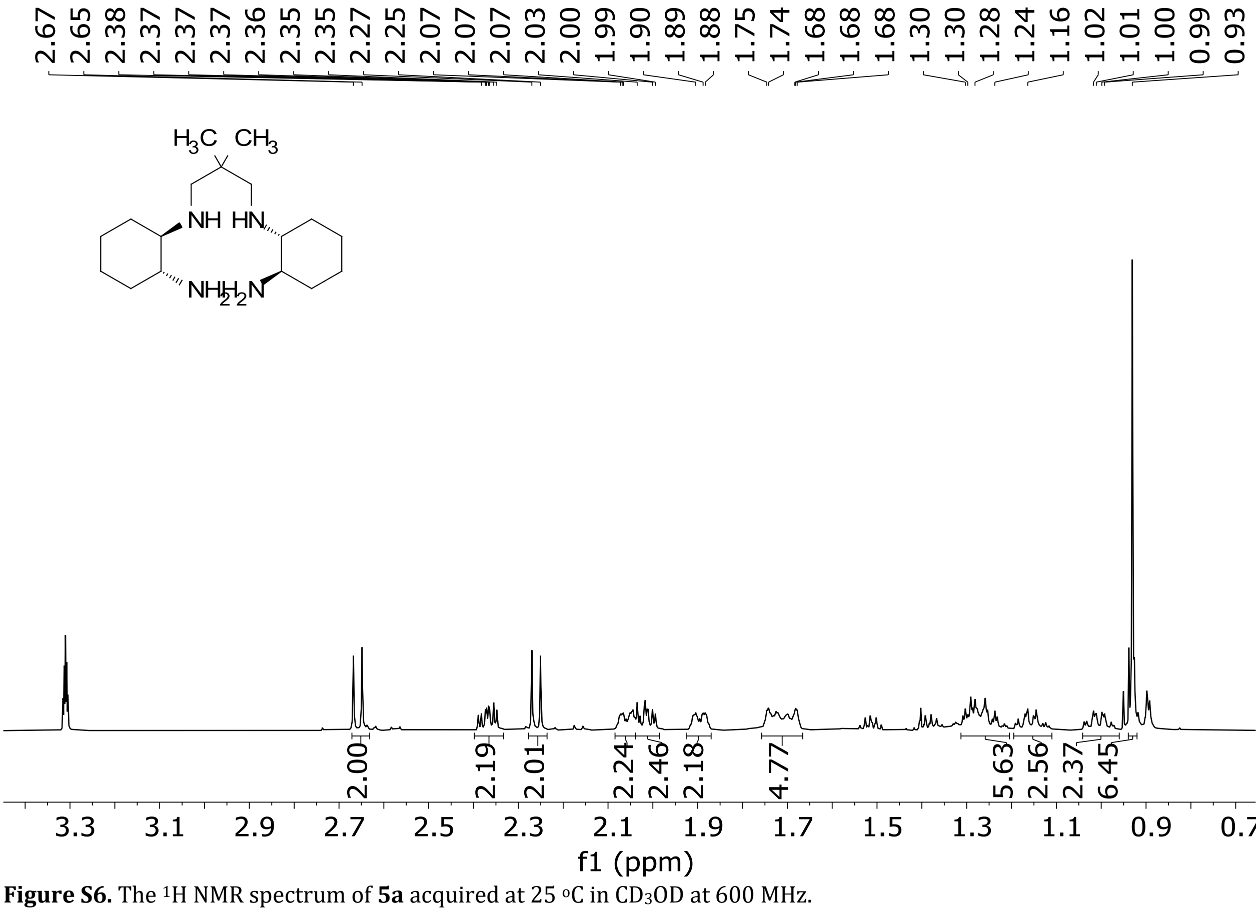

Figure S6. The ^{1}H NMR spectrum of **5a** acquired at 25 °C in CD_3OD at 600 MHz.

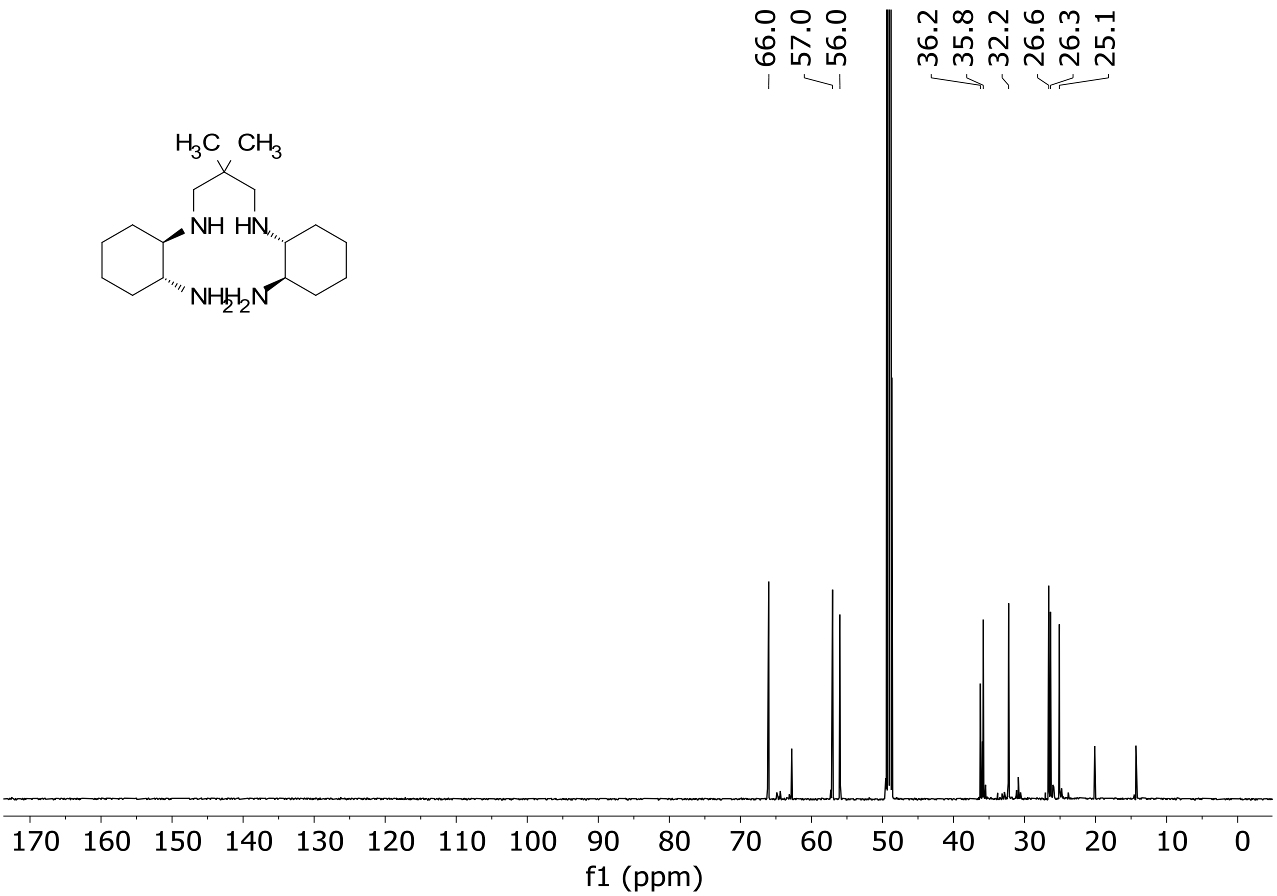

Figure S7. The ^{13}C NMR spectrum of **5a** acquired at 25 °C in CD_3OD at 151 MHz.

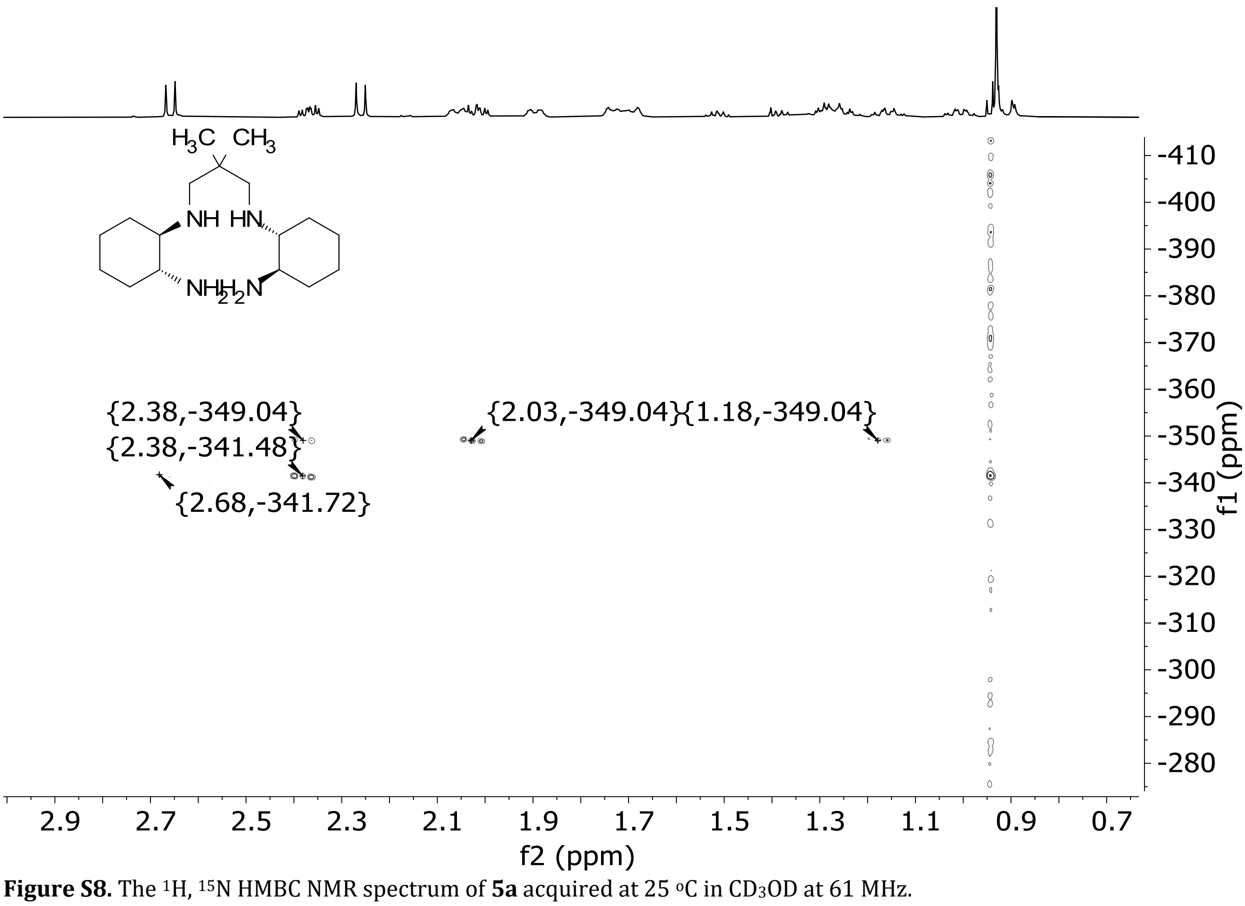

Figure S8. The ^{1}H, ^{15}N HMBC NMR spectrum of **5a** acquired at 25 °C in CD_3OD at 61 MHz.

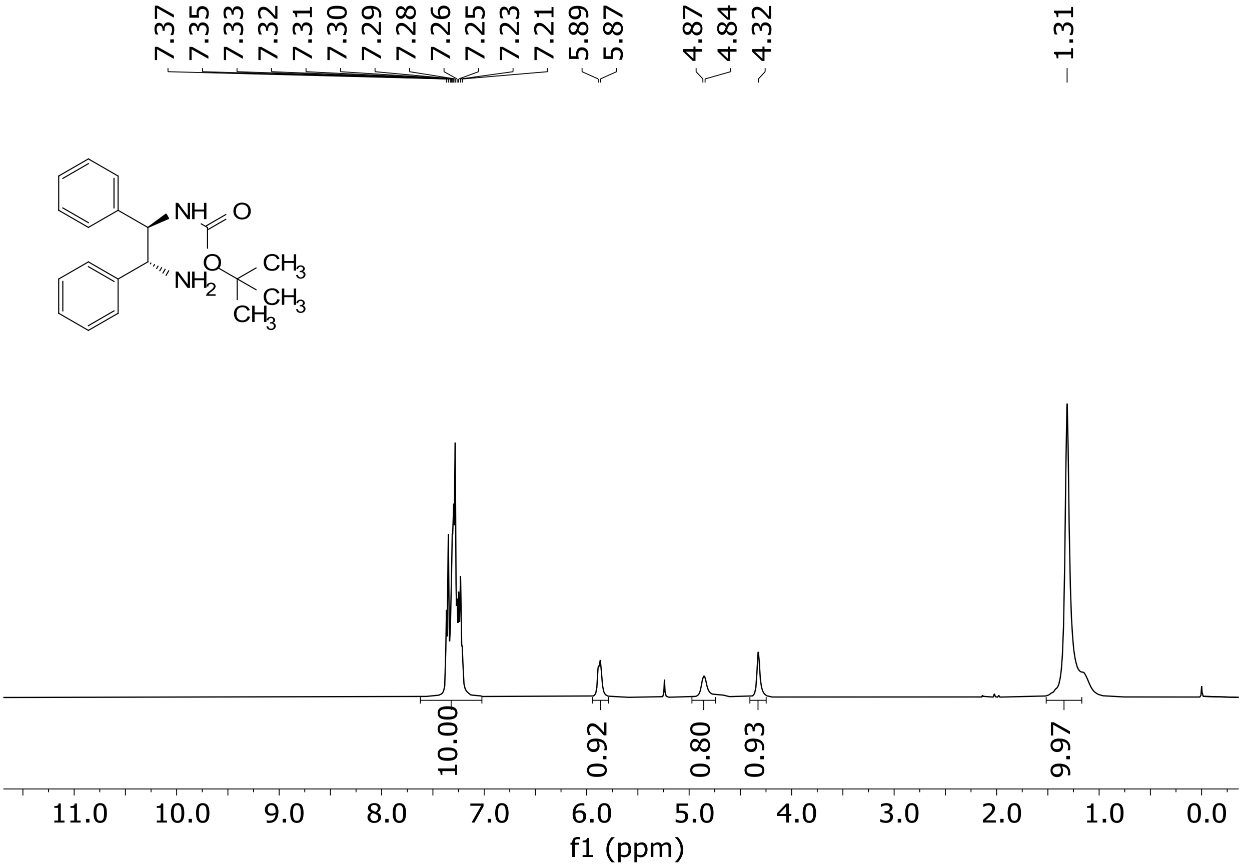

Figure S9. The ^{1}H NMR spectrum of **B**-boc acquired at 25 °C in $CDCl_3$ at 400 MHz.

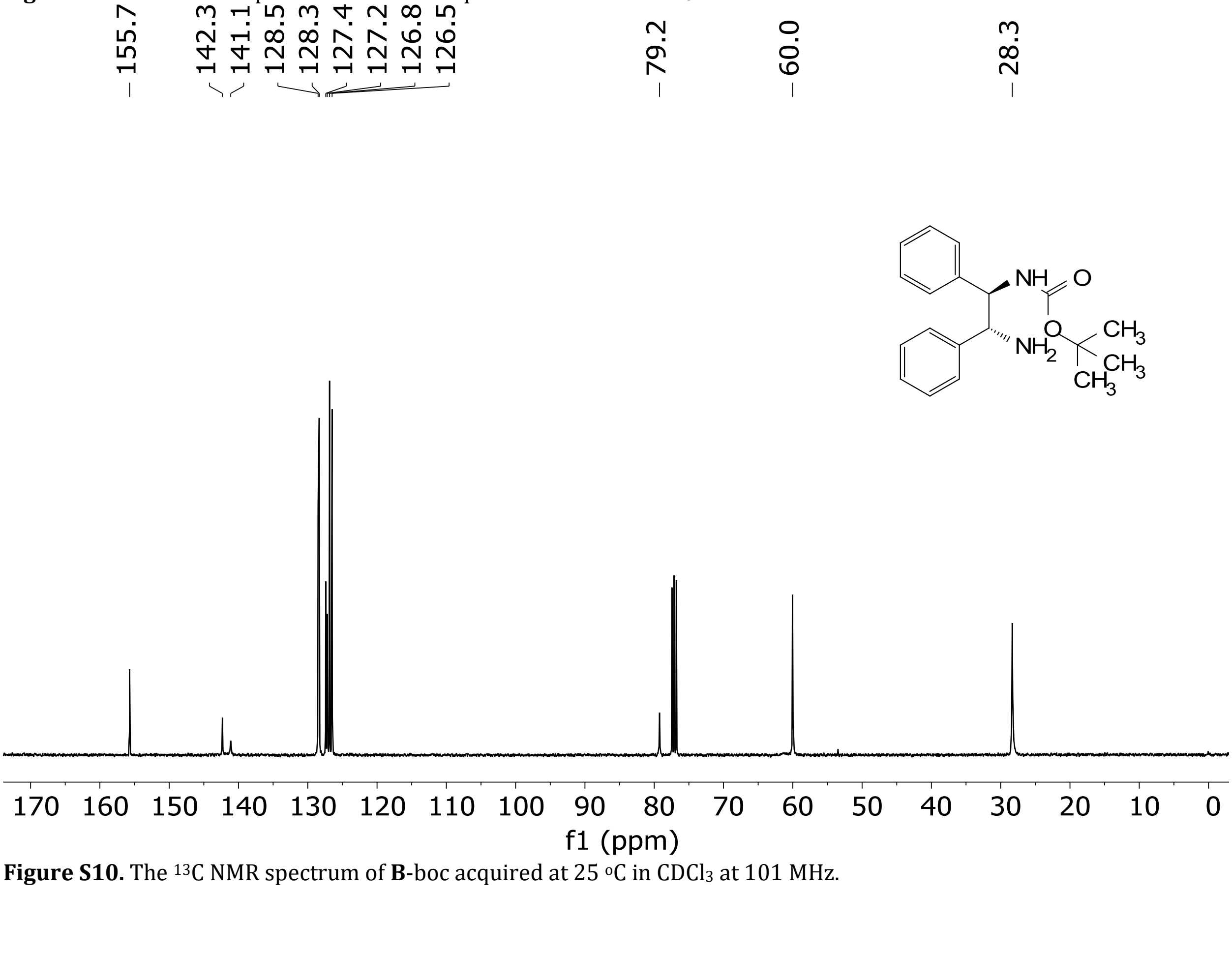

Figure S10. The ^{13}C NMR spectrum of **B**-boc acquired at 25 °C in $CDCl_3$ at 101 MHz.

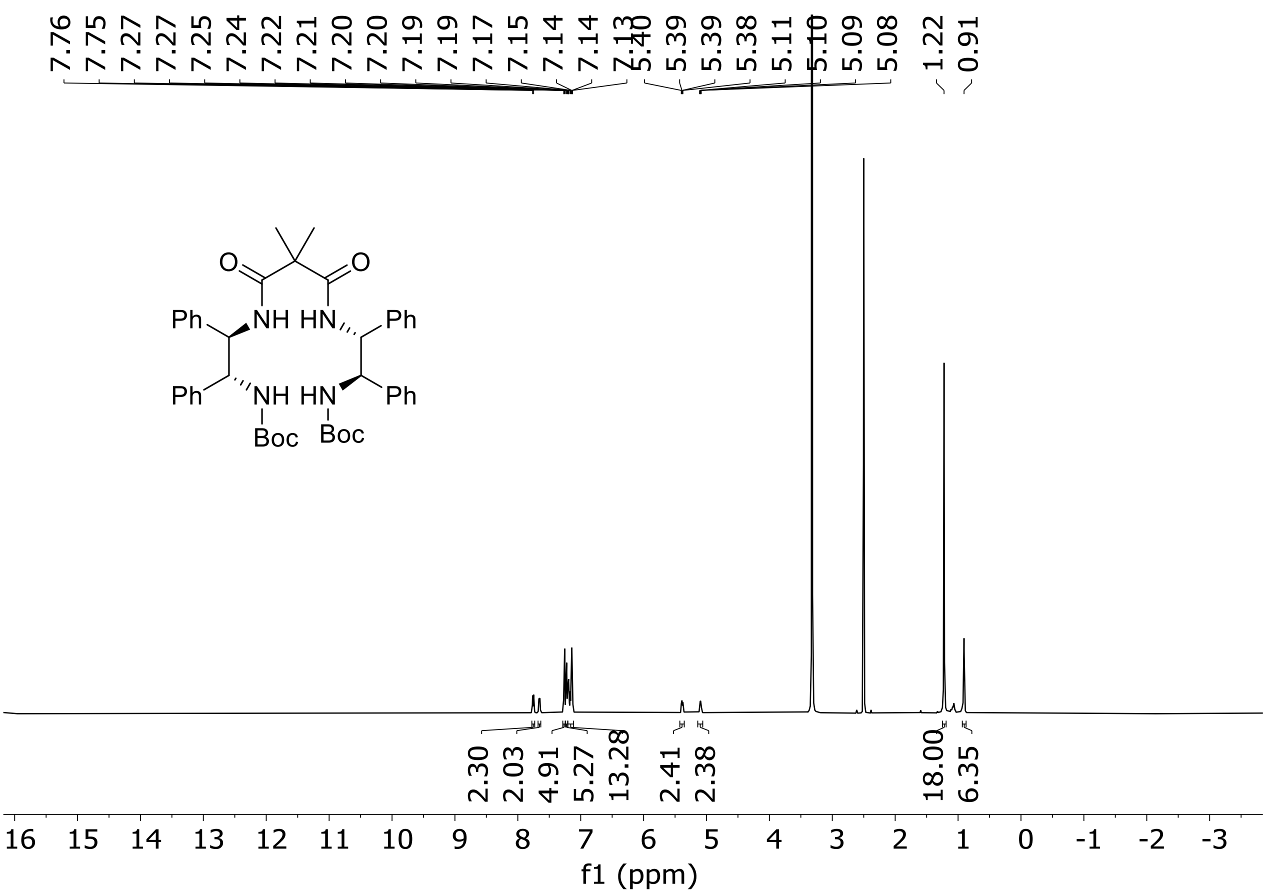

Figure S11. The ^{1}H NMR spectrum of **1b**-boc acquired at 25 °C in d_6-DMSO at 600 MHz.

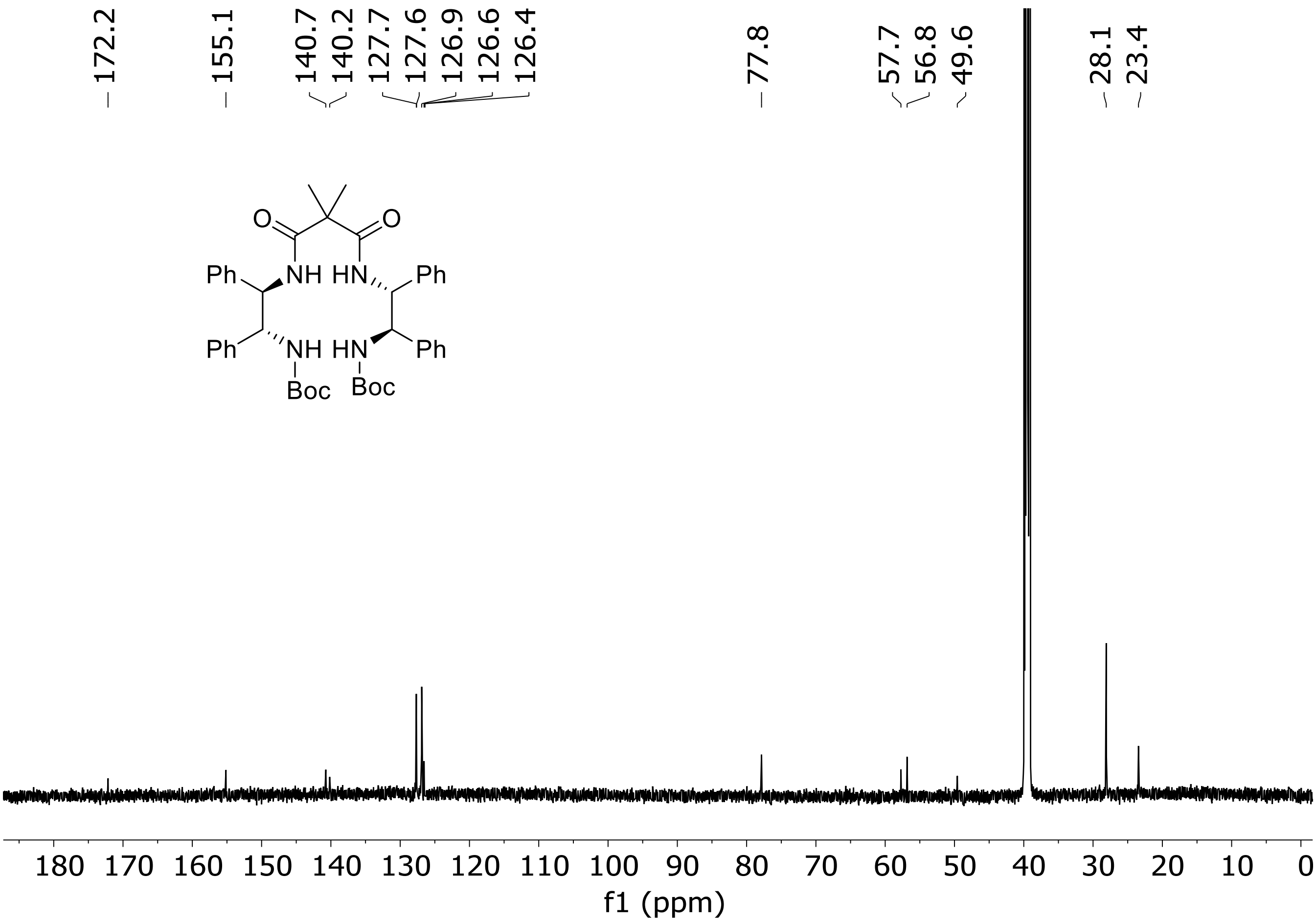

Figure S12. The ^{13}C NMR spectrum of **1b**-boc acquired at 25 °C in d_6-DMSO at 151 MHz.

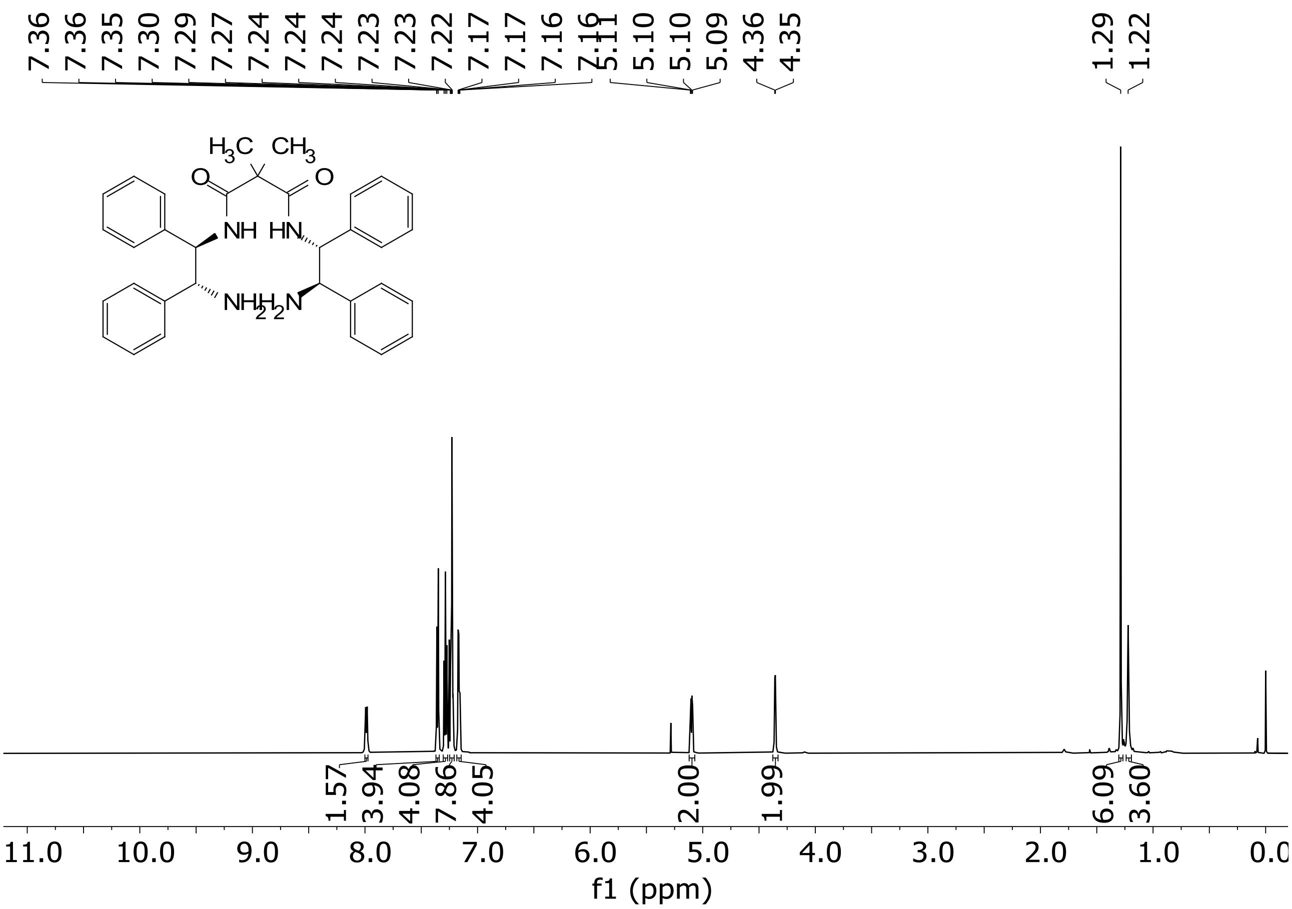

Figure S13. The ^{1}H NMR spectrum of **1b** acquired at 25 °C in $CDCl_3$ at 600 MHz.

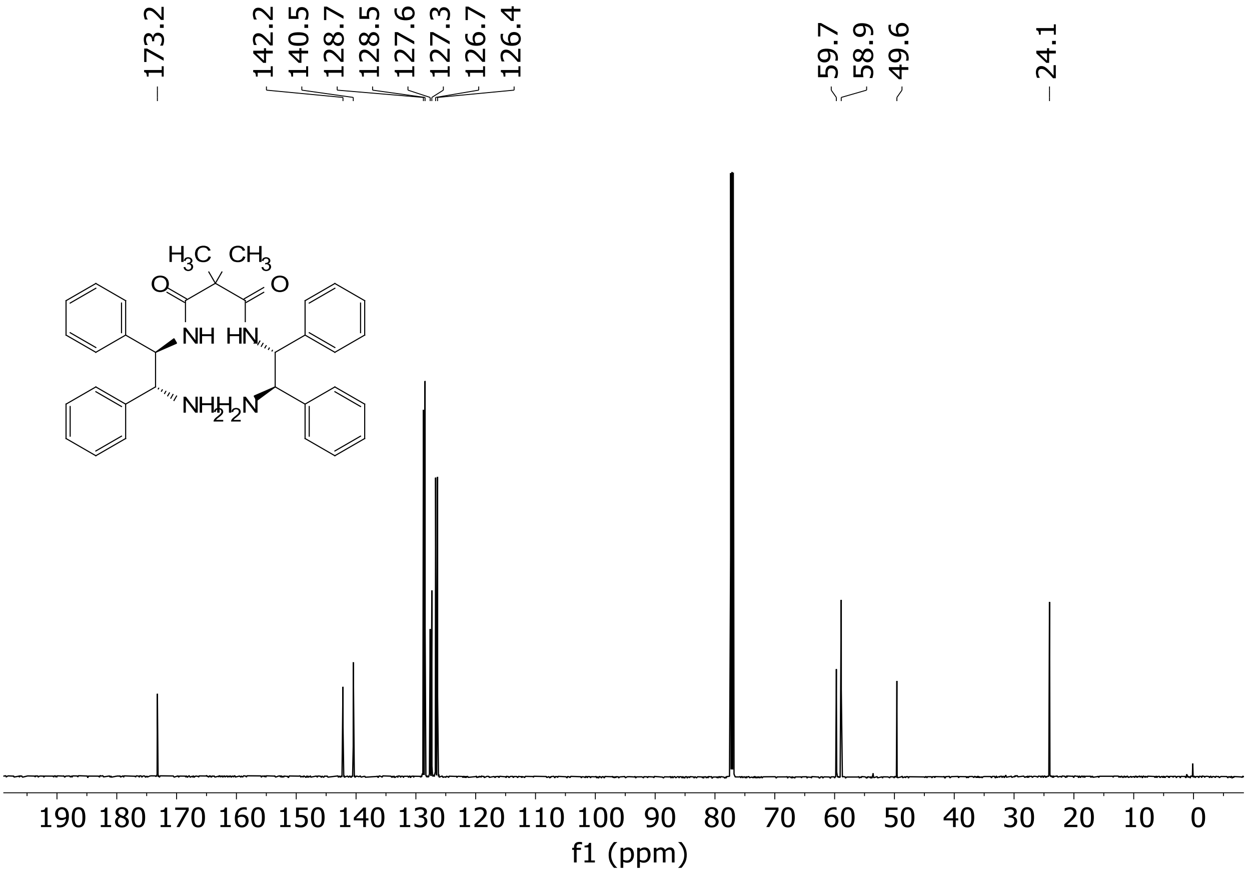

Figure S14. The ^{13}C NMR spectrum of **1b** acquired at 25 °C in $CDCl_3$ at 151 MHz.

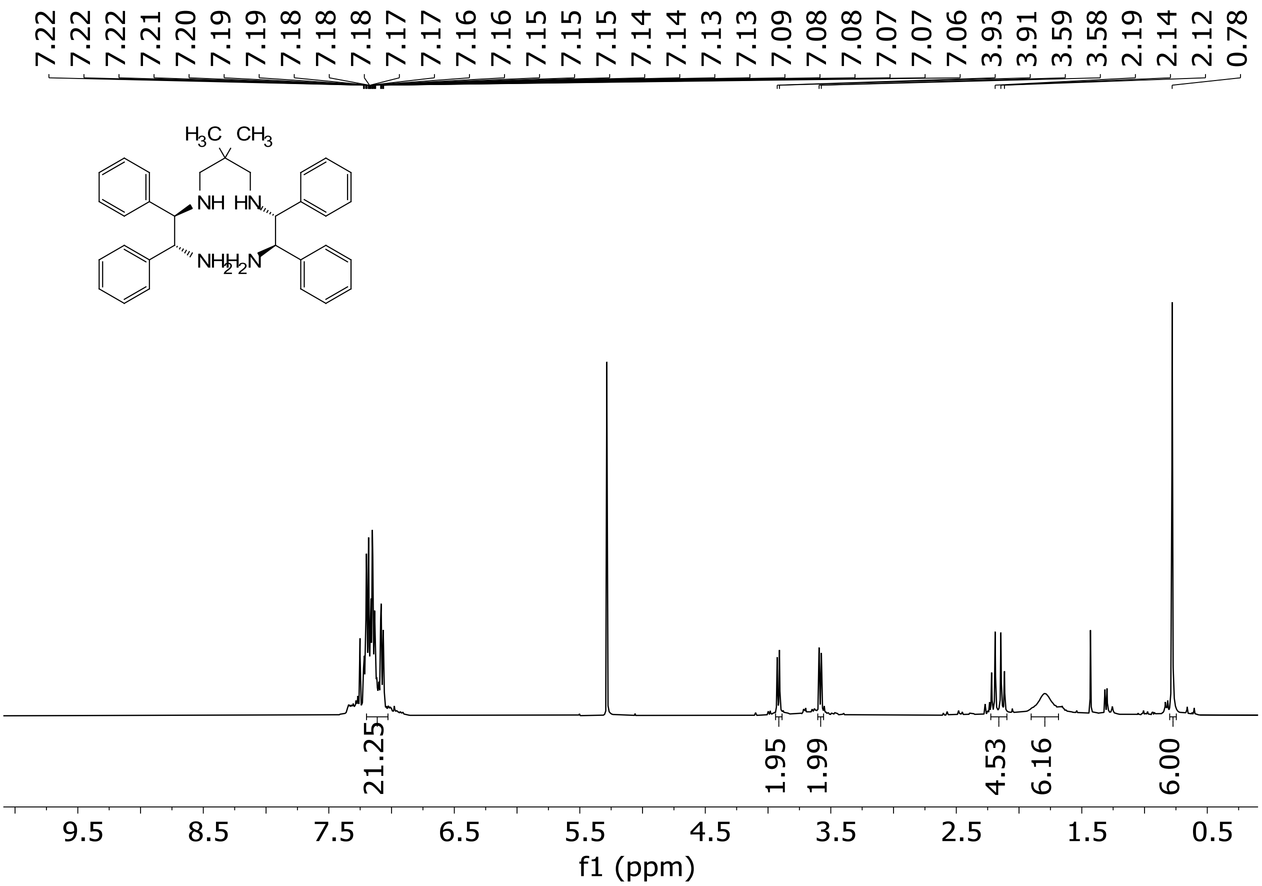

Figure S15. The ^{1}H NMR spectrum of **5b** acquired at 25 °C in $CDCl_3$ at 400 MHz.

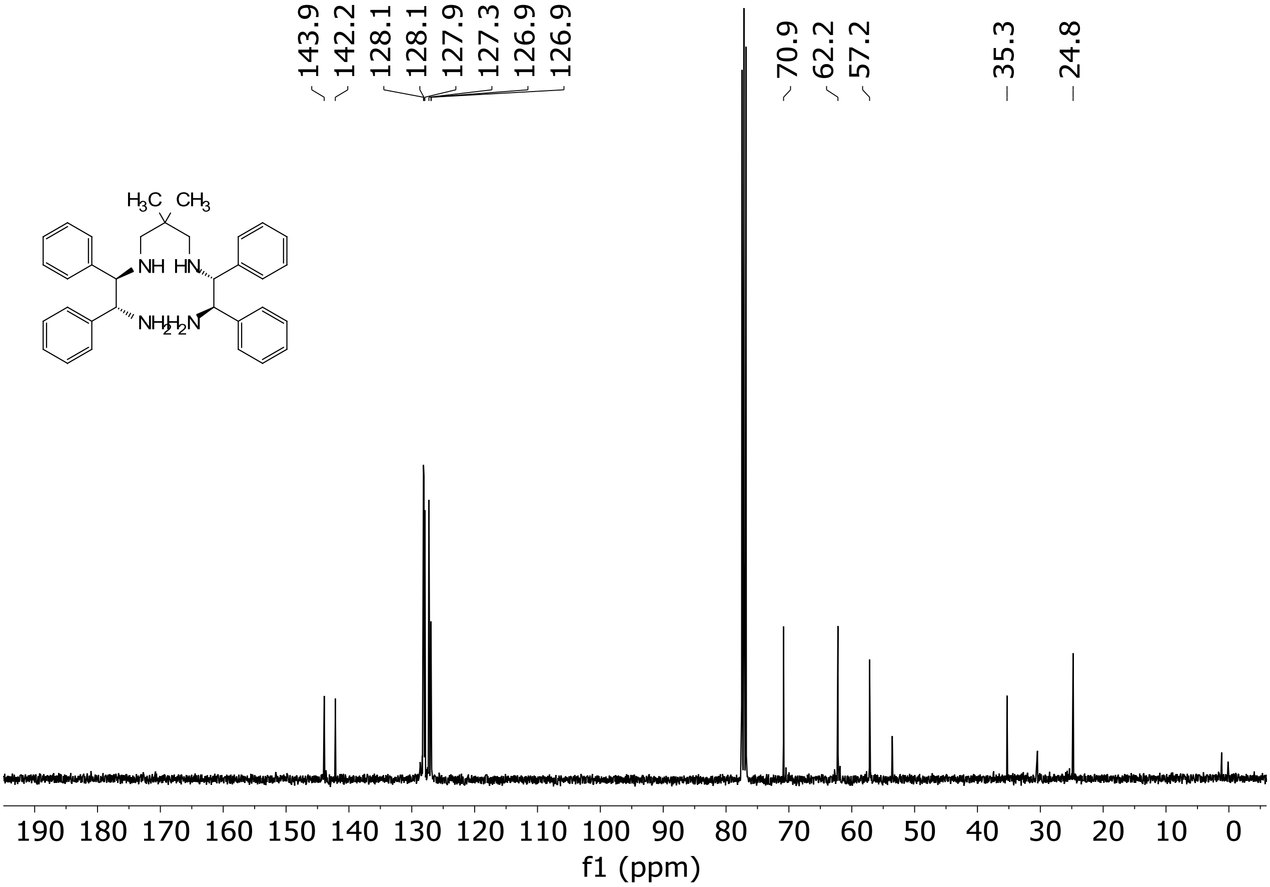

Figure S16. The ^{13}C NMR spectrum of **5b** acquired at 25 °C in $CDCl_3$ at 101 MHz.

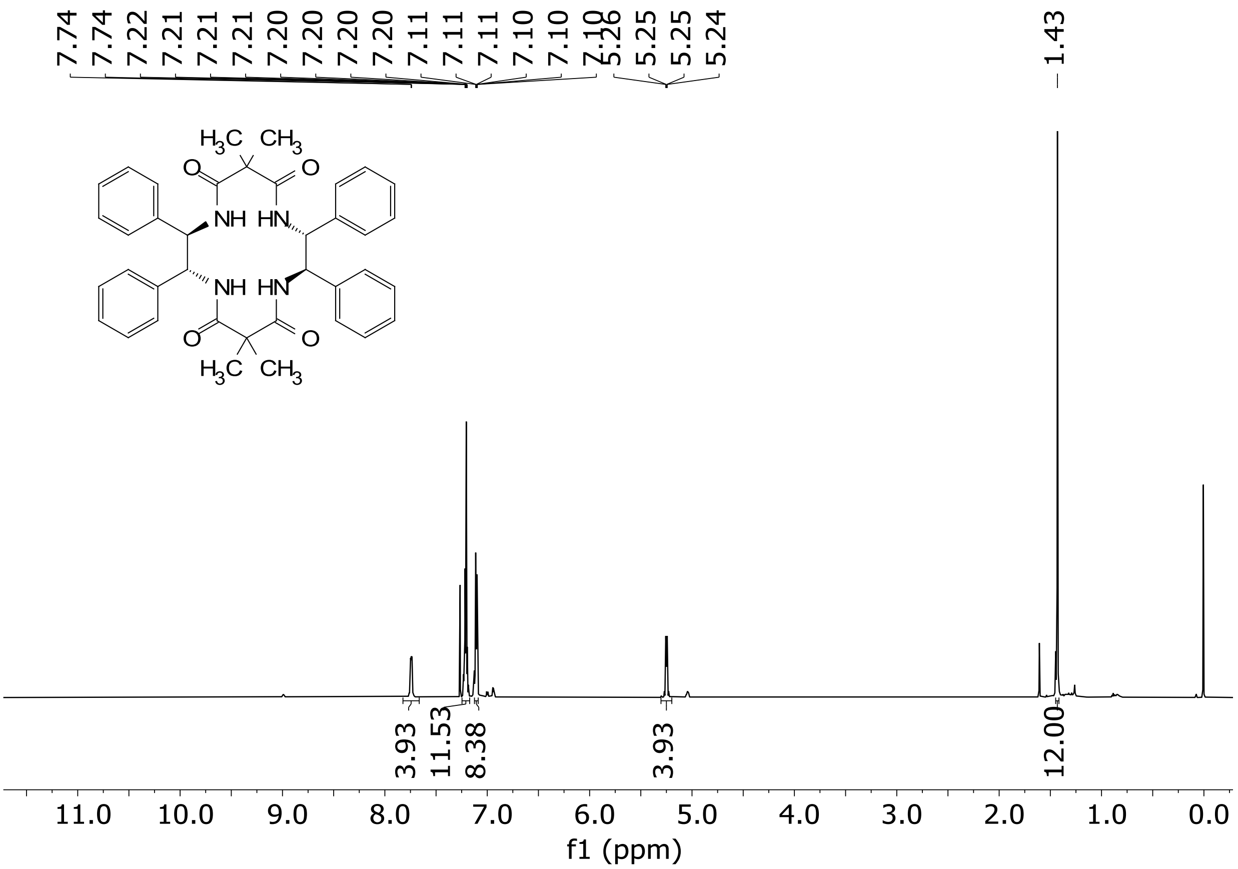

Figure S17. The ^{1}H NMR spectrum of **2b** acquired at 25 °C in $CDCl_3$ at 600 MHz.

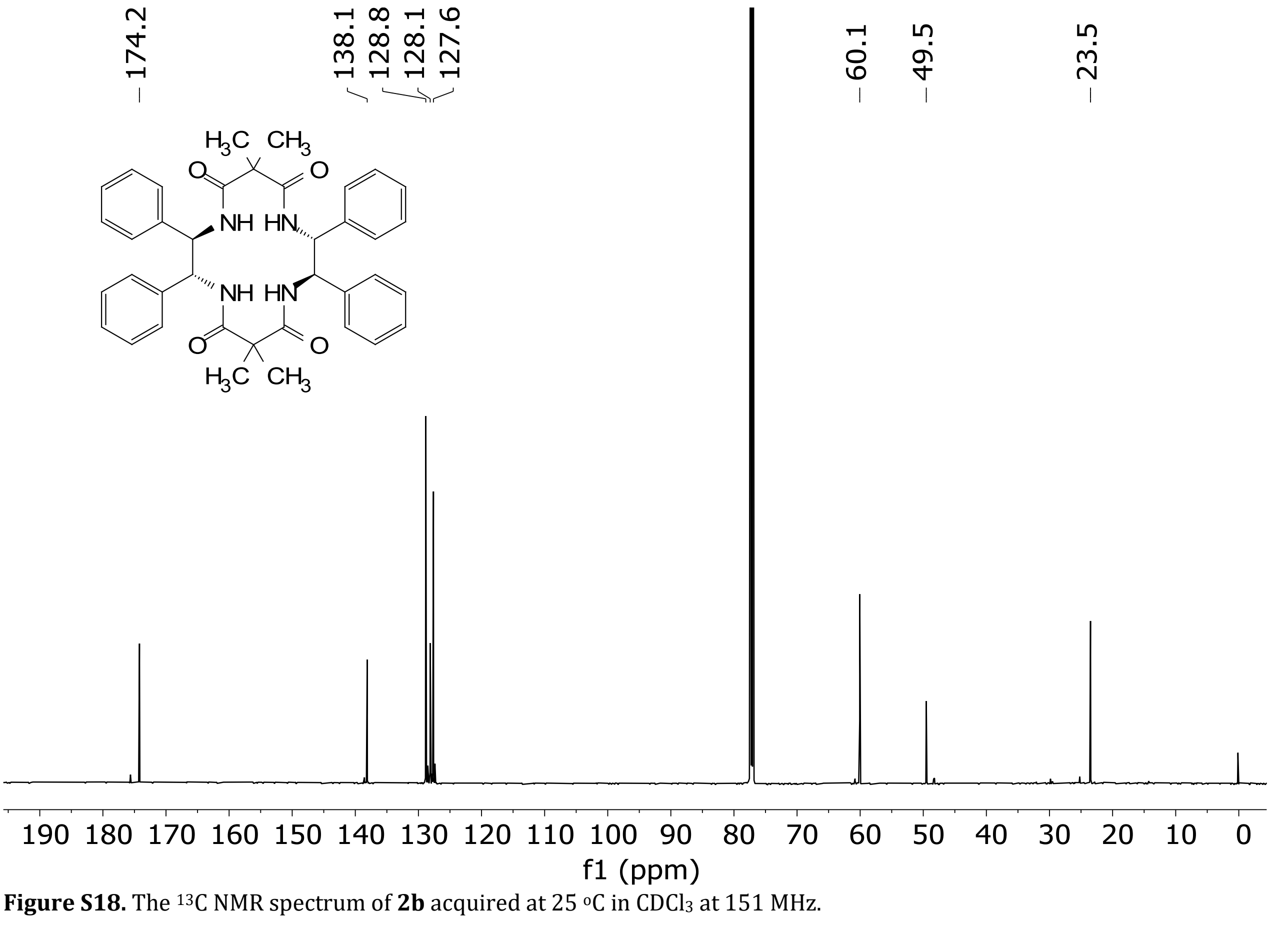

Figure S18. The ^{13}C NMR spectrum of **2b** acquired at 25 °C in $CDCl_3$ at 151 MHz.

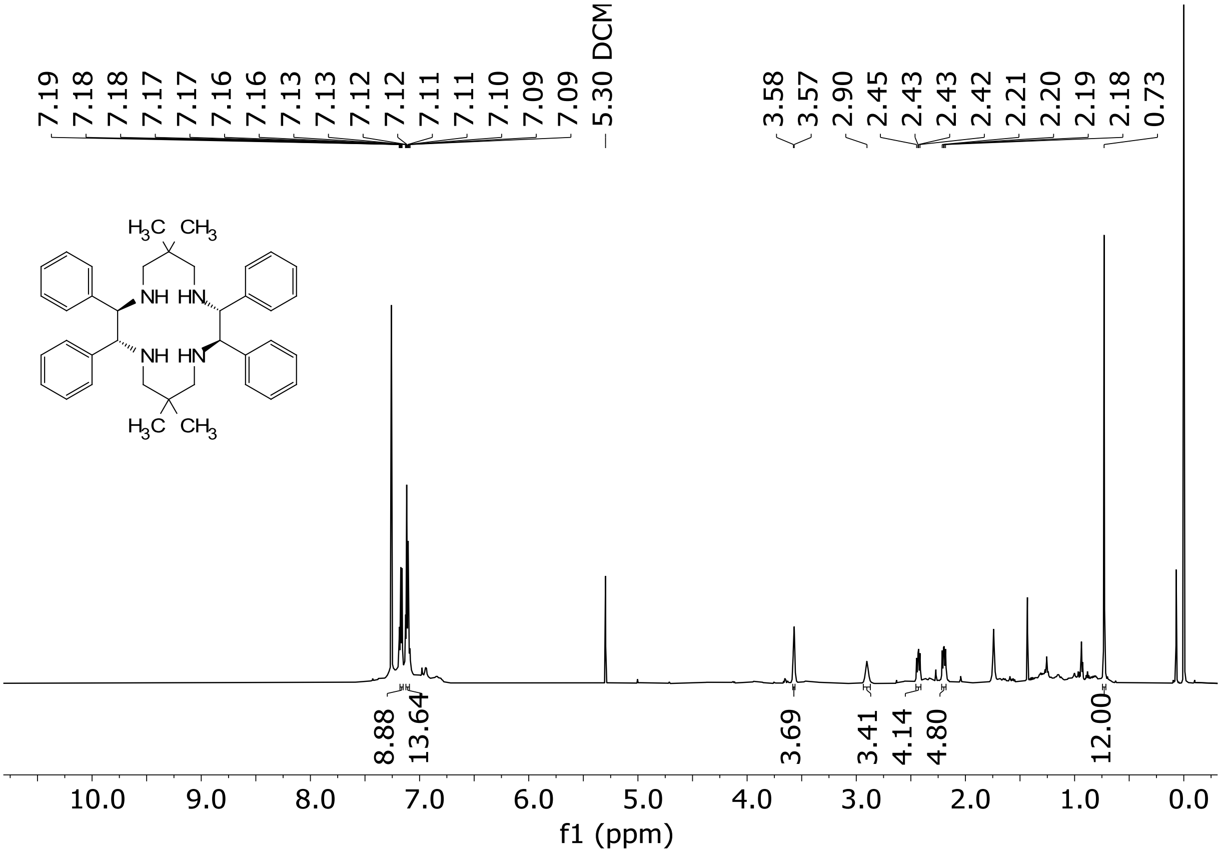

Figure S19. The ^{1}H NMR spectrum of **6b** acquired at 25 °C in $CDCl_3$ at 600 MHz.

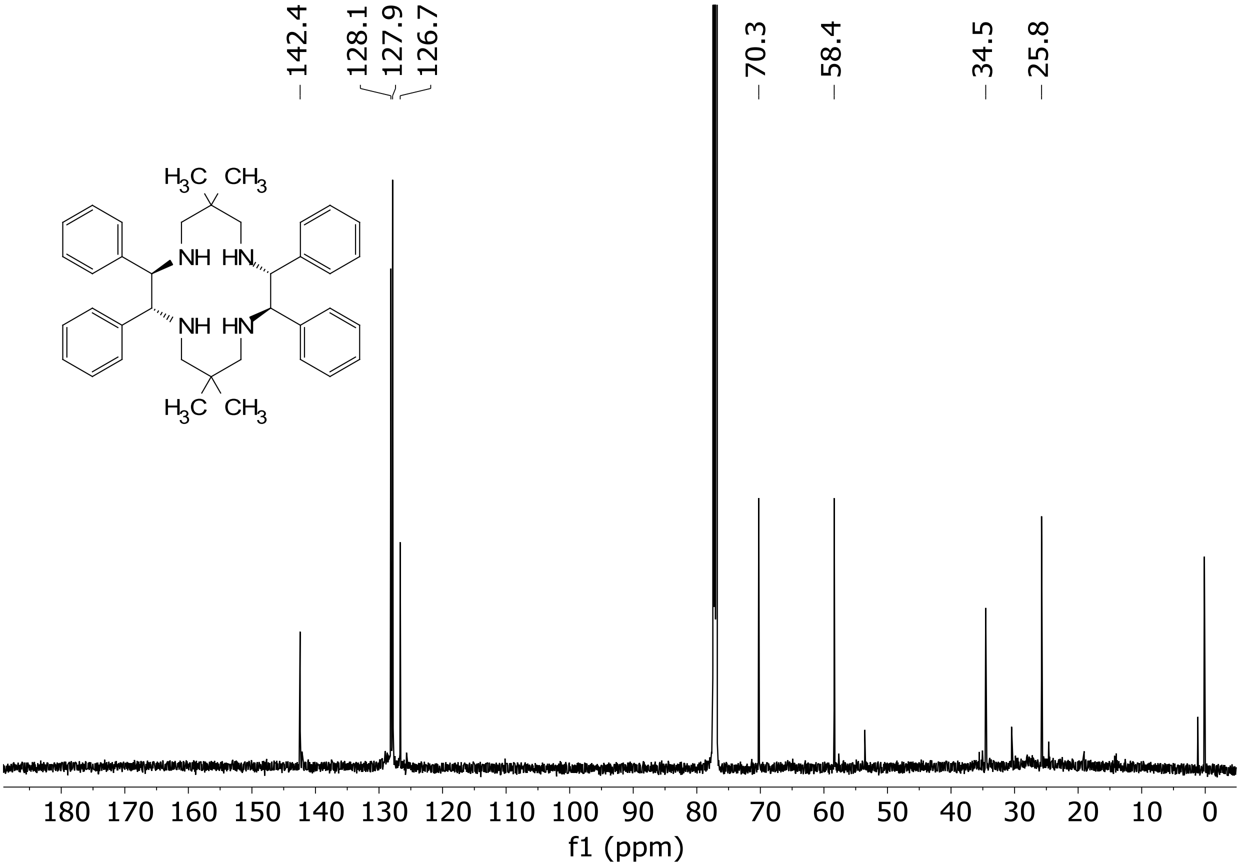

Figure S20. The ^{13}C NMR spectrum of **6b** acquired at 25 °C in $CDCl_3$ at 151 MHz.

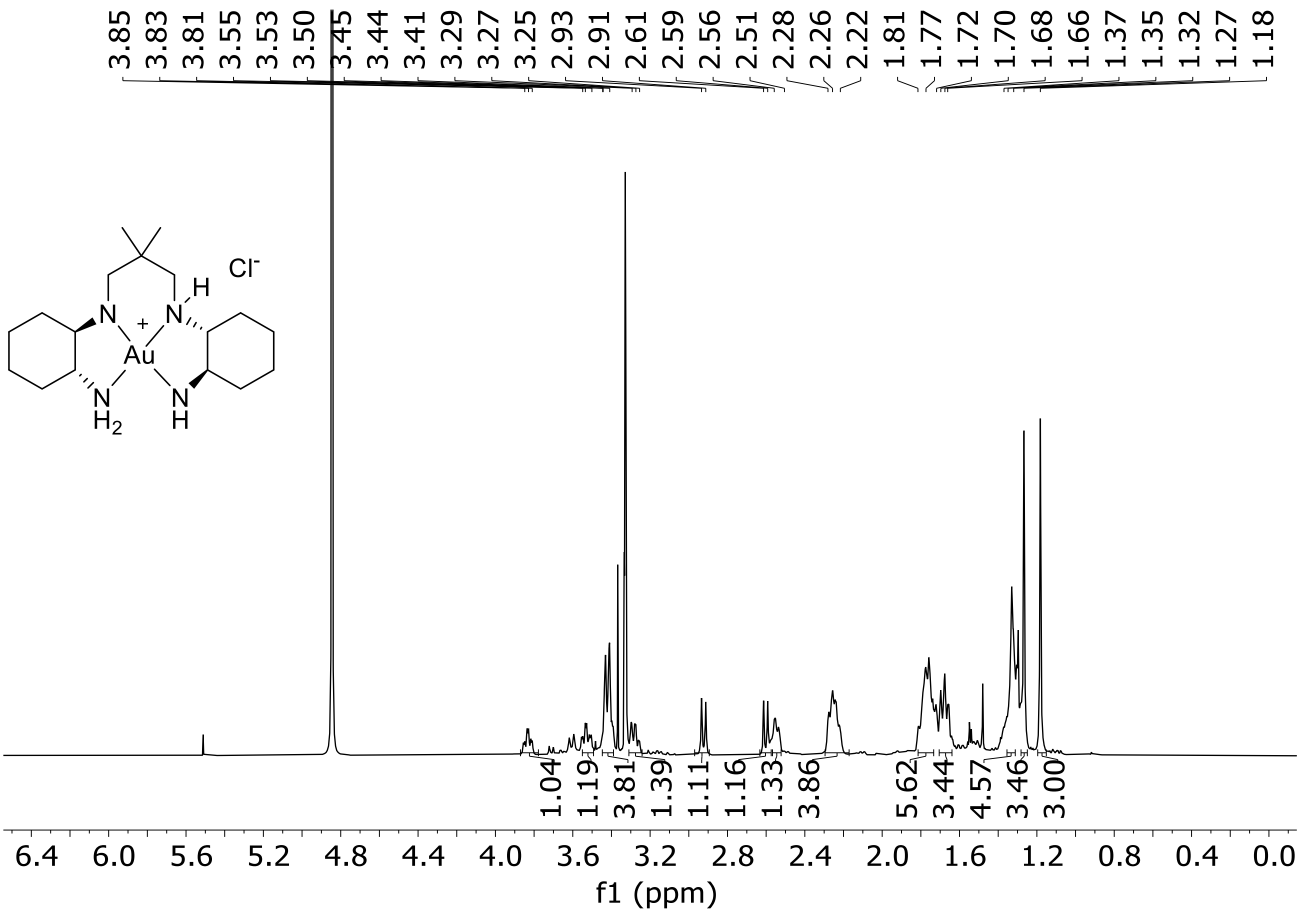

Figure S21. The ^{1}H NMR spectrum of **5a**-Au(III) acquired at 25 °C in CD_3OD at 600 MHz.

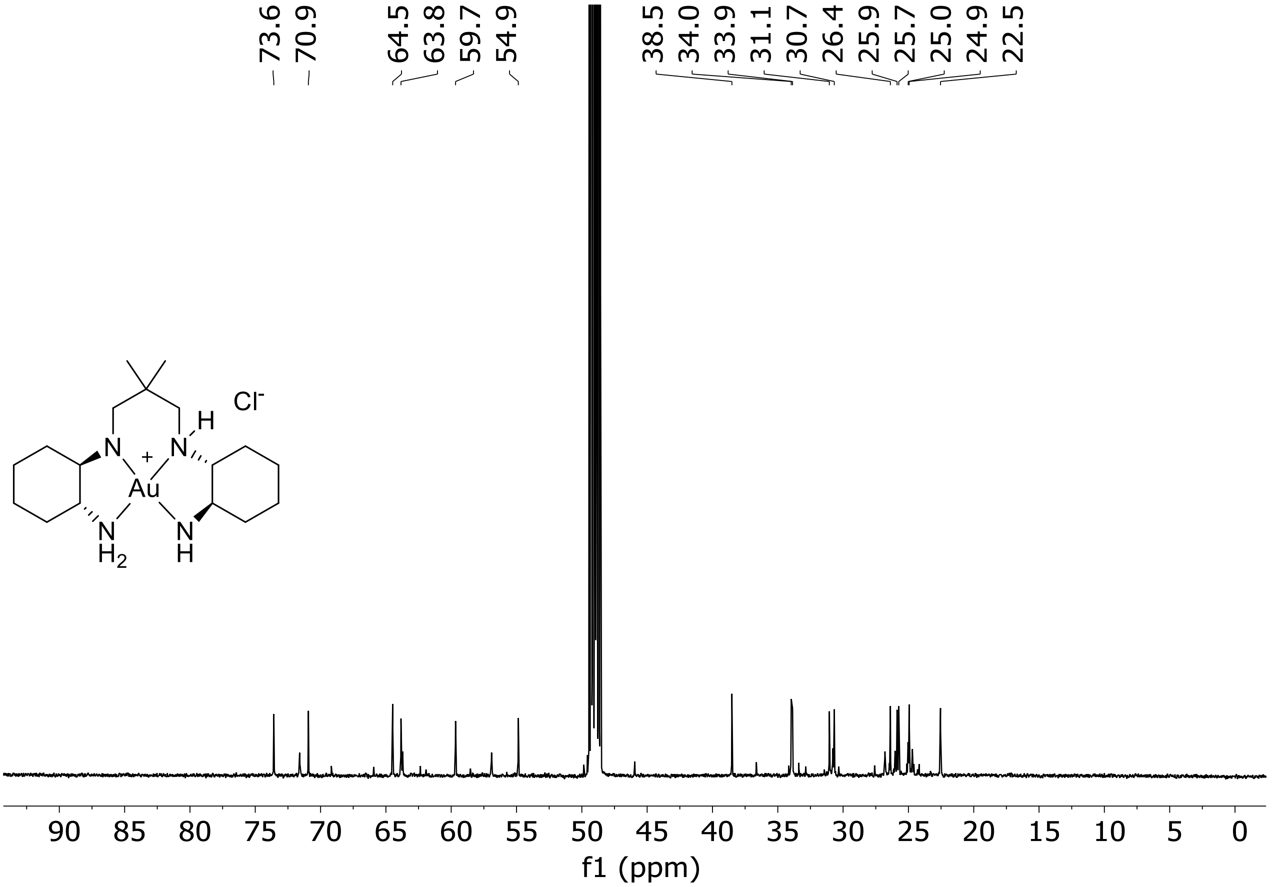

Figure S22. The ^{13}C NMR spectrum of **5a**-Au(III) acquired at 25 °C in CD_3OD at 151 MHz

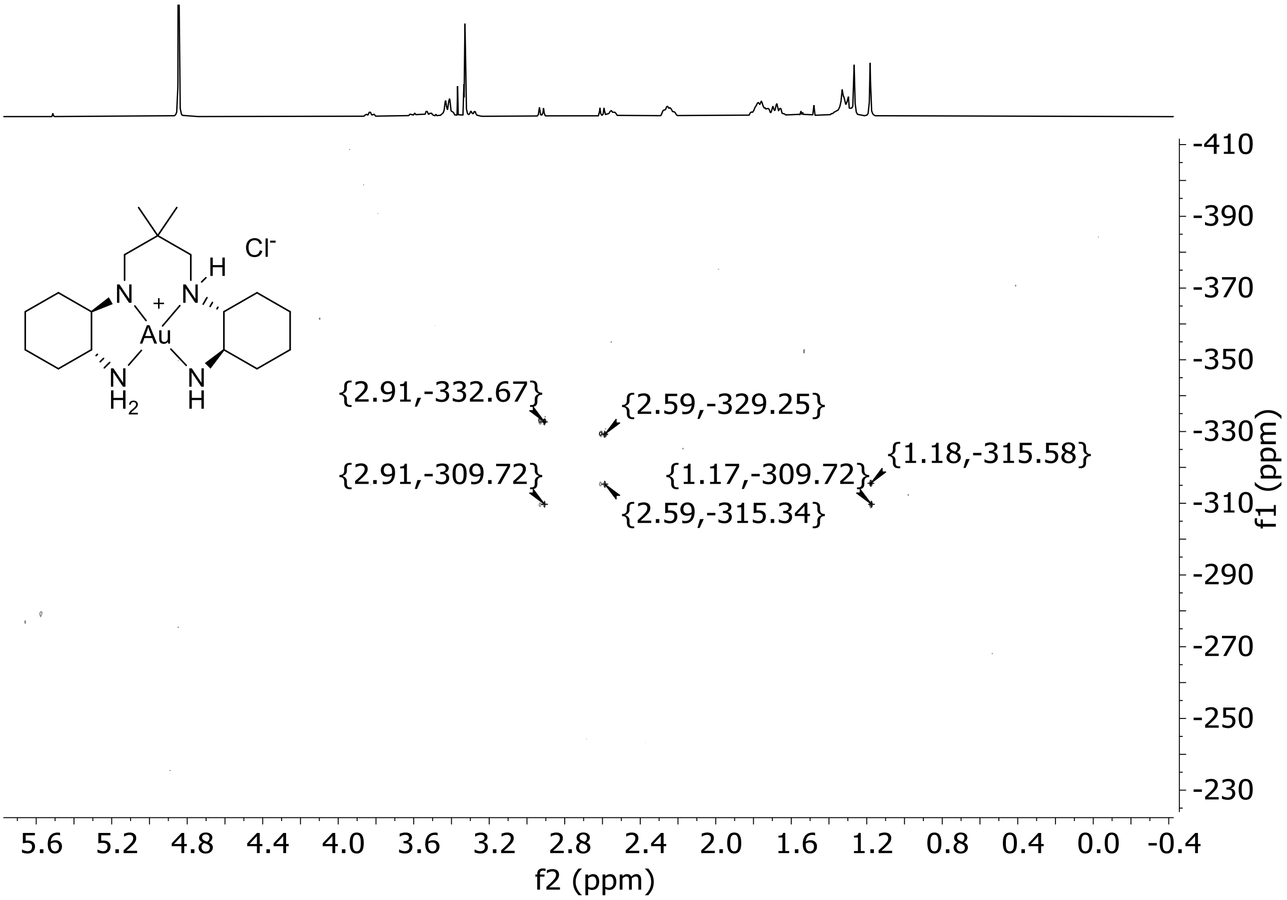

Figure S23. The ^{1}H, ^{15}N HMBC NMR spectrum of **5a**-Au(III) acquired at 25 °C in CD_3OD at 61 MHz.

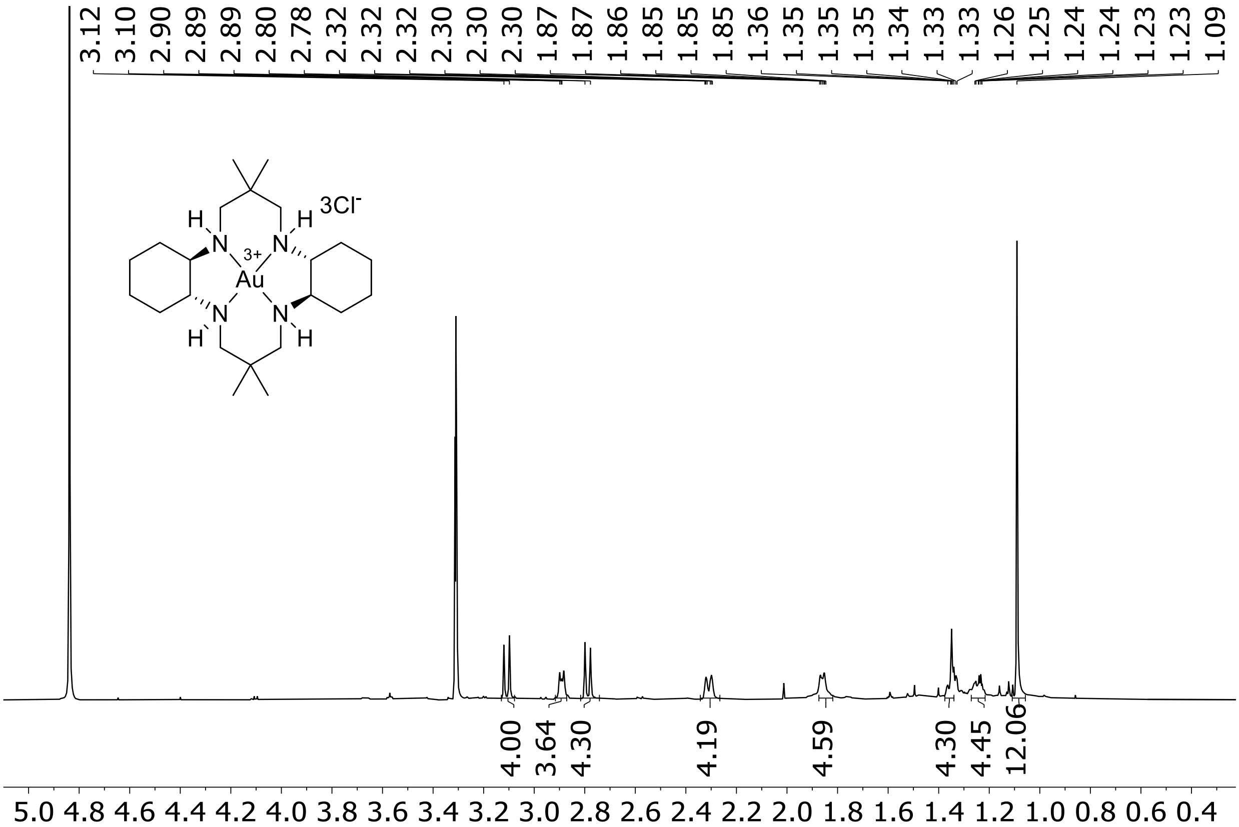

Figure S24. The ^{1}H NMR spectrum of **6a**-Au(III) acquired at 25 °C in CD_3OD at 600 MHz.

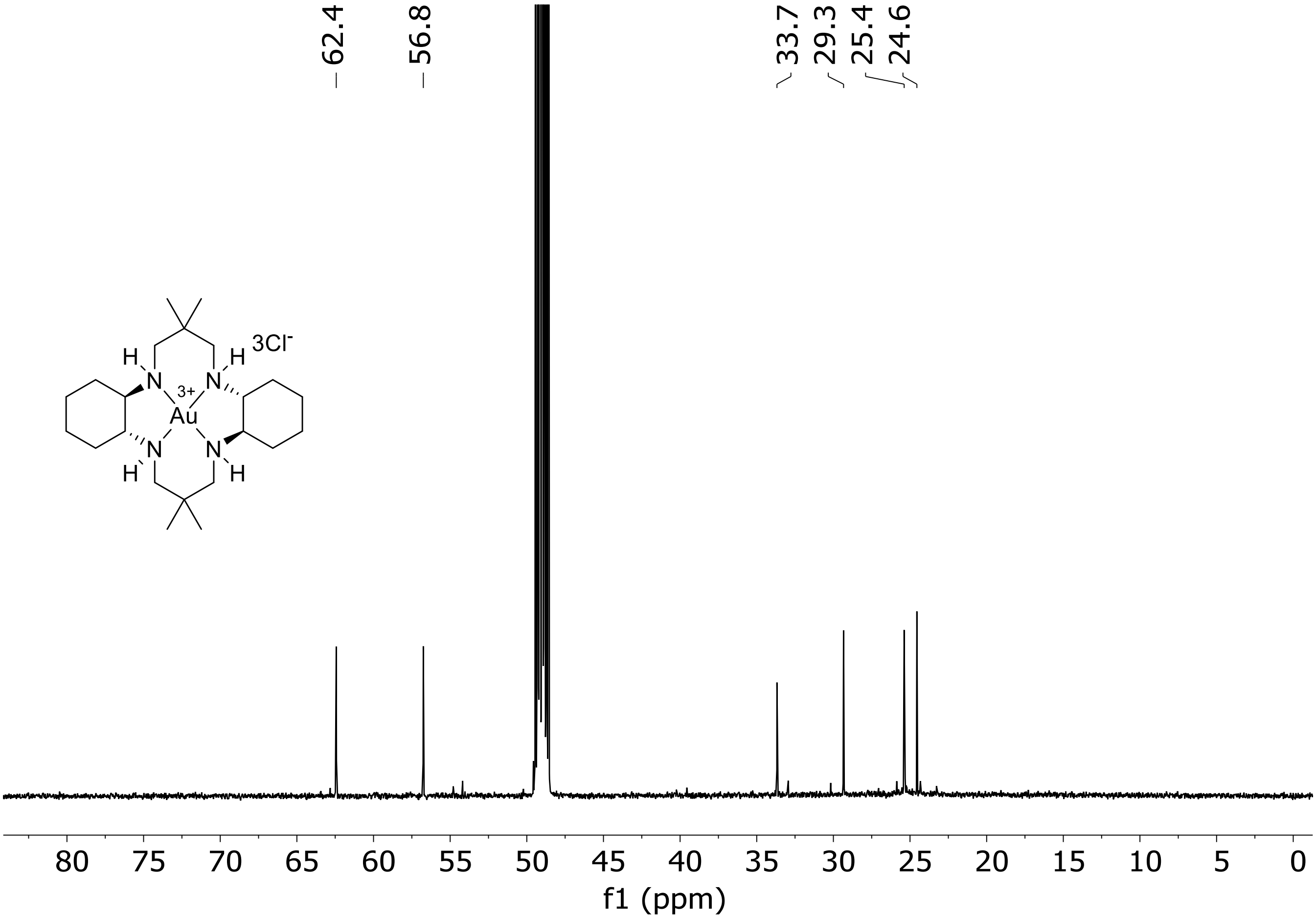

Figure S25. The ^{13}C NMR spectrum of **6a**-Au(III) acquired at 25 °C in CD_3OD at 151 MHz

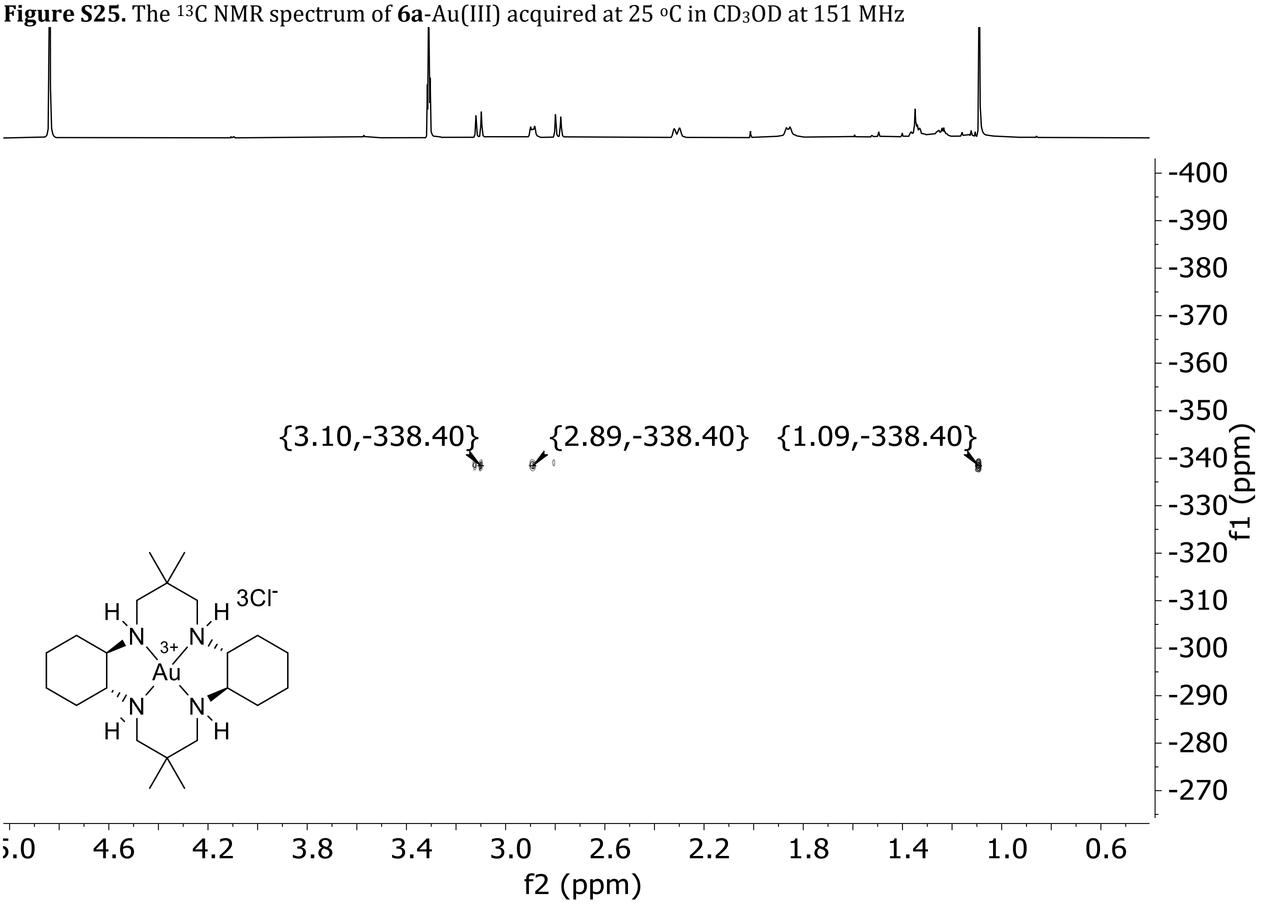

Figure S26. The ^{1}H, ^{15}N HMBC NMR spectrum of **6a**-Au(III) acquired at 25 °C in CD_3OD at 61 MHz.

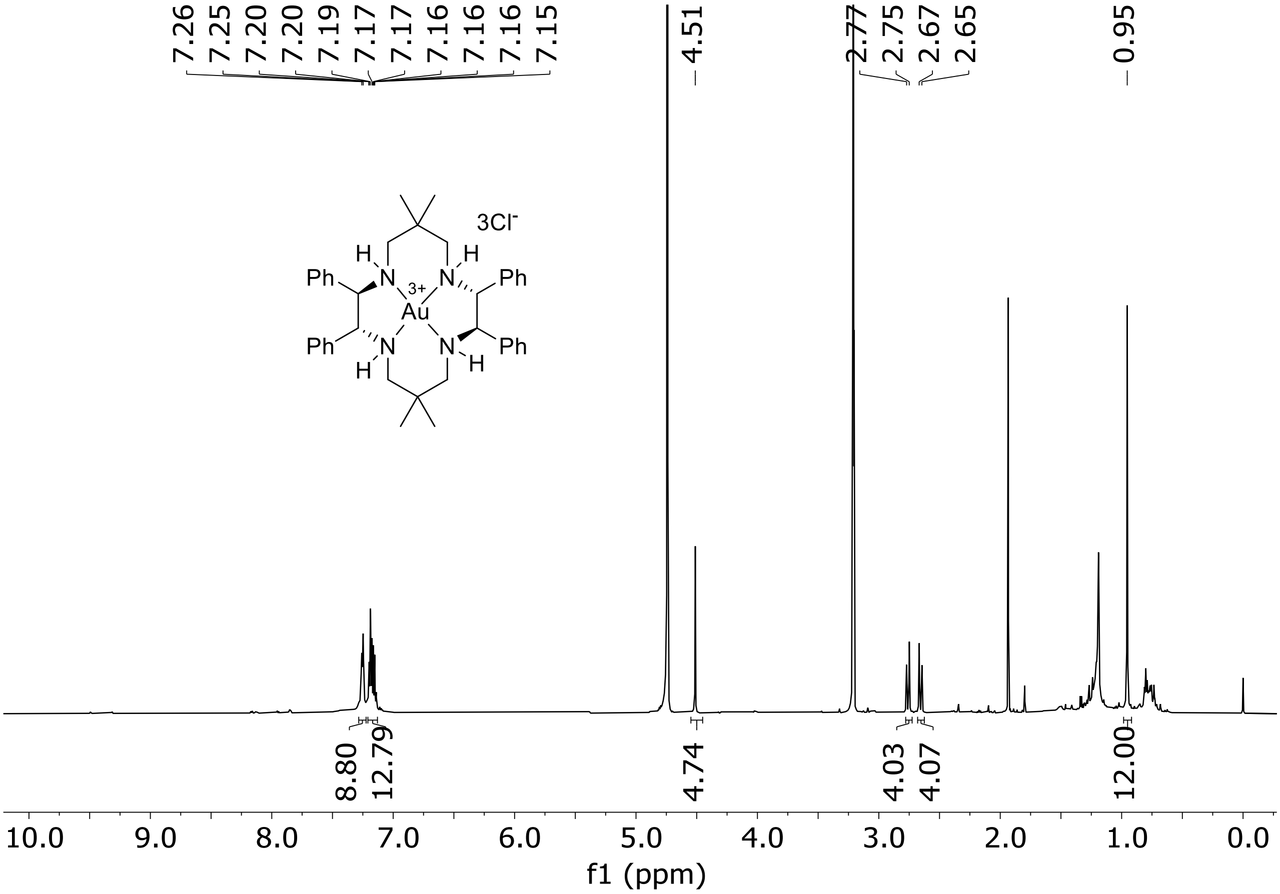

Figure S27. The ^{1}H NMR spectrum of **6b**-Au(III) acquired at 25 °C in CD_3OD at 600 MHz.

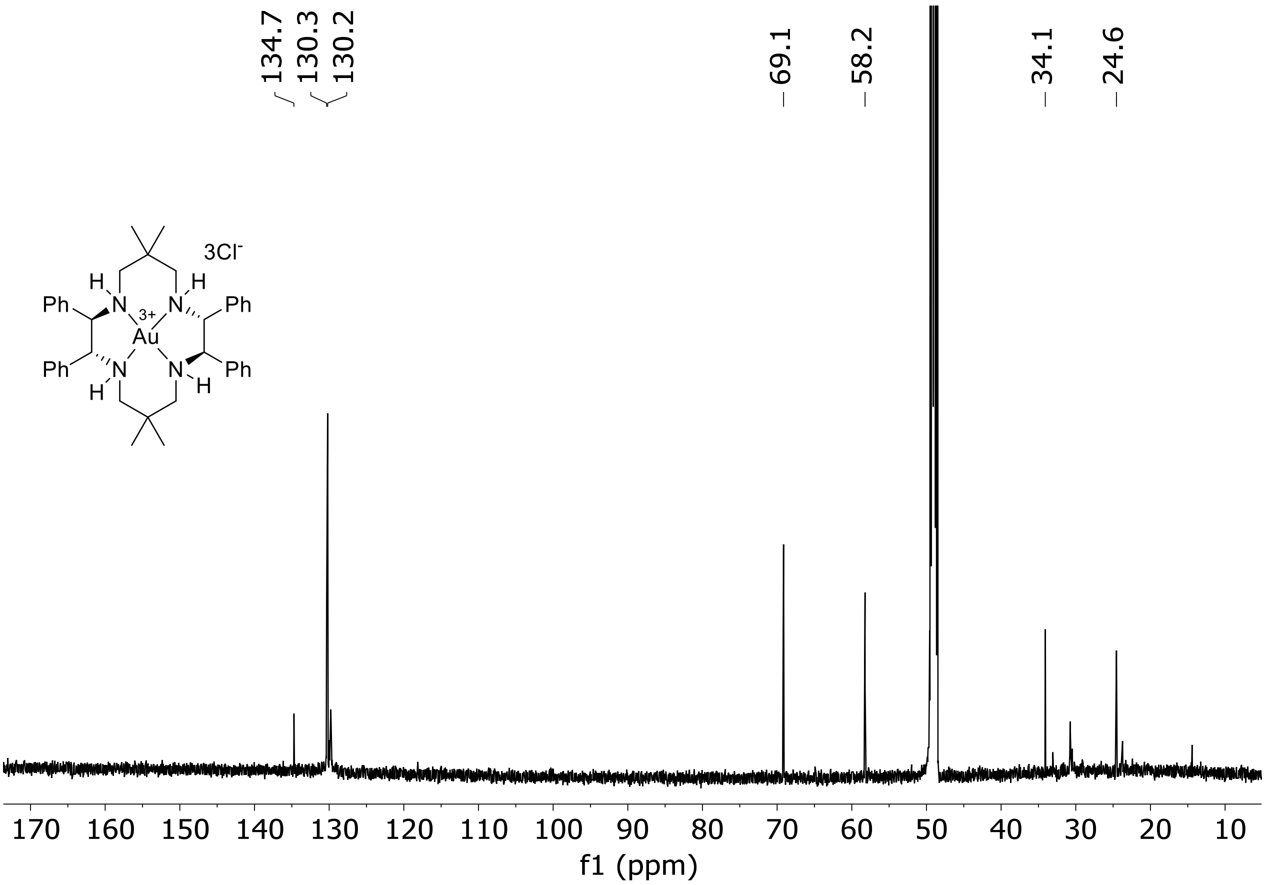

Figure S28. The ^{13}C NMR spectrum of **6b**-Au(III) acquired at 25 °C in CD_3OD at 151 MHz

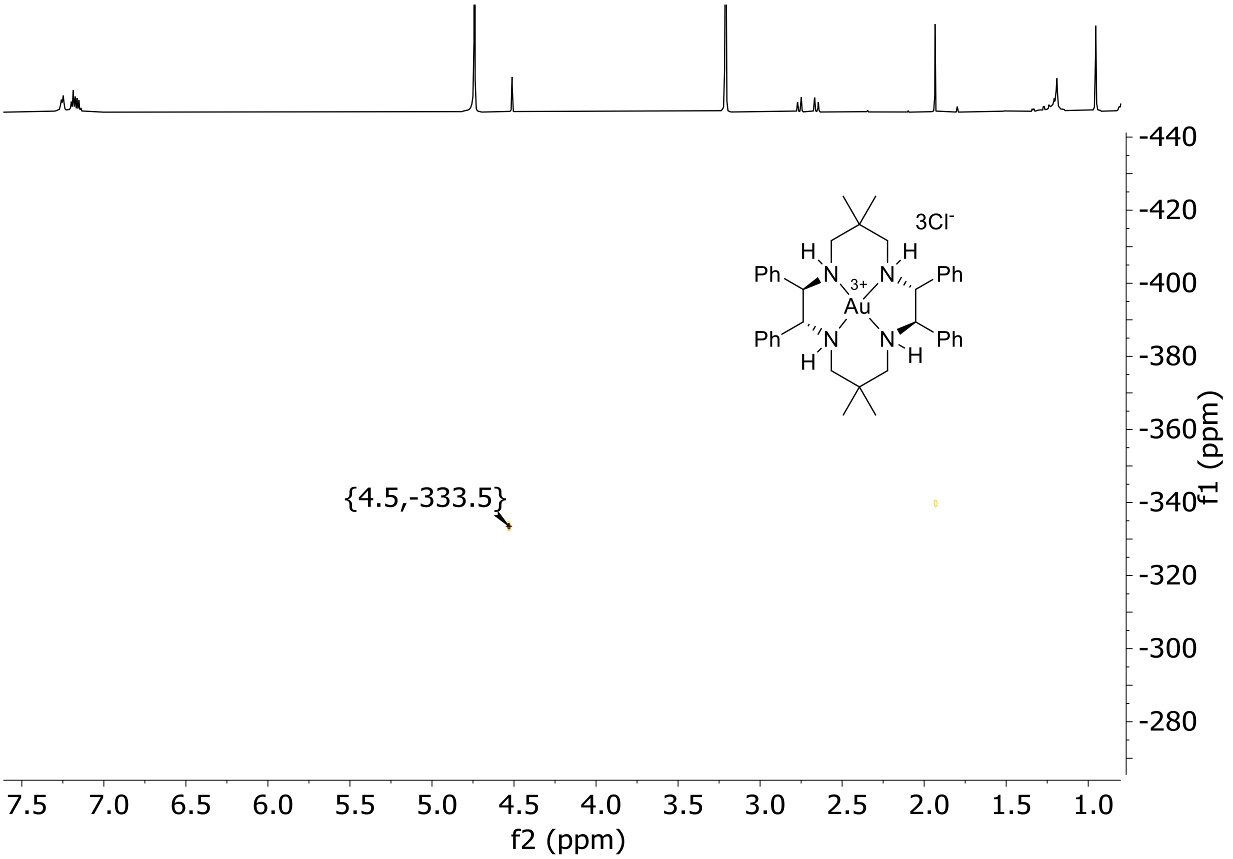

Figure S29. The ^{1}H, ^{15}N HMBC NMR spectrum of **6b**-Au(III) acquired at 25 °C in CD_3OD at 61 MHz.

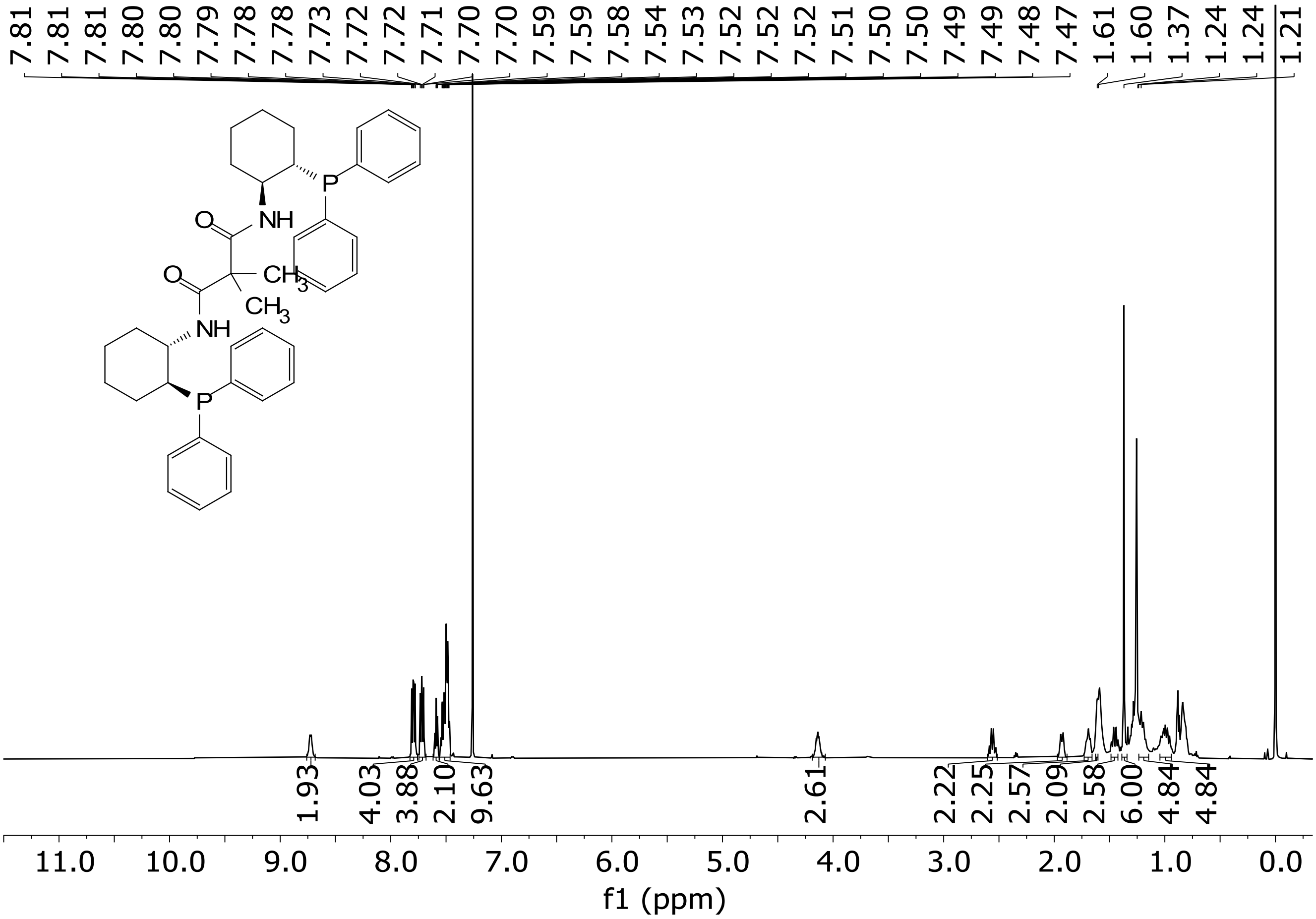

Figure S30. The ^{1}H NMR spectrum of **1c** acquired at 25 °C in $CDCl_3$ at 600 MHz.

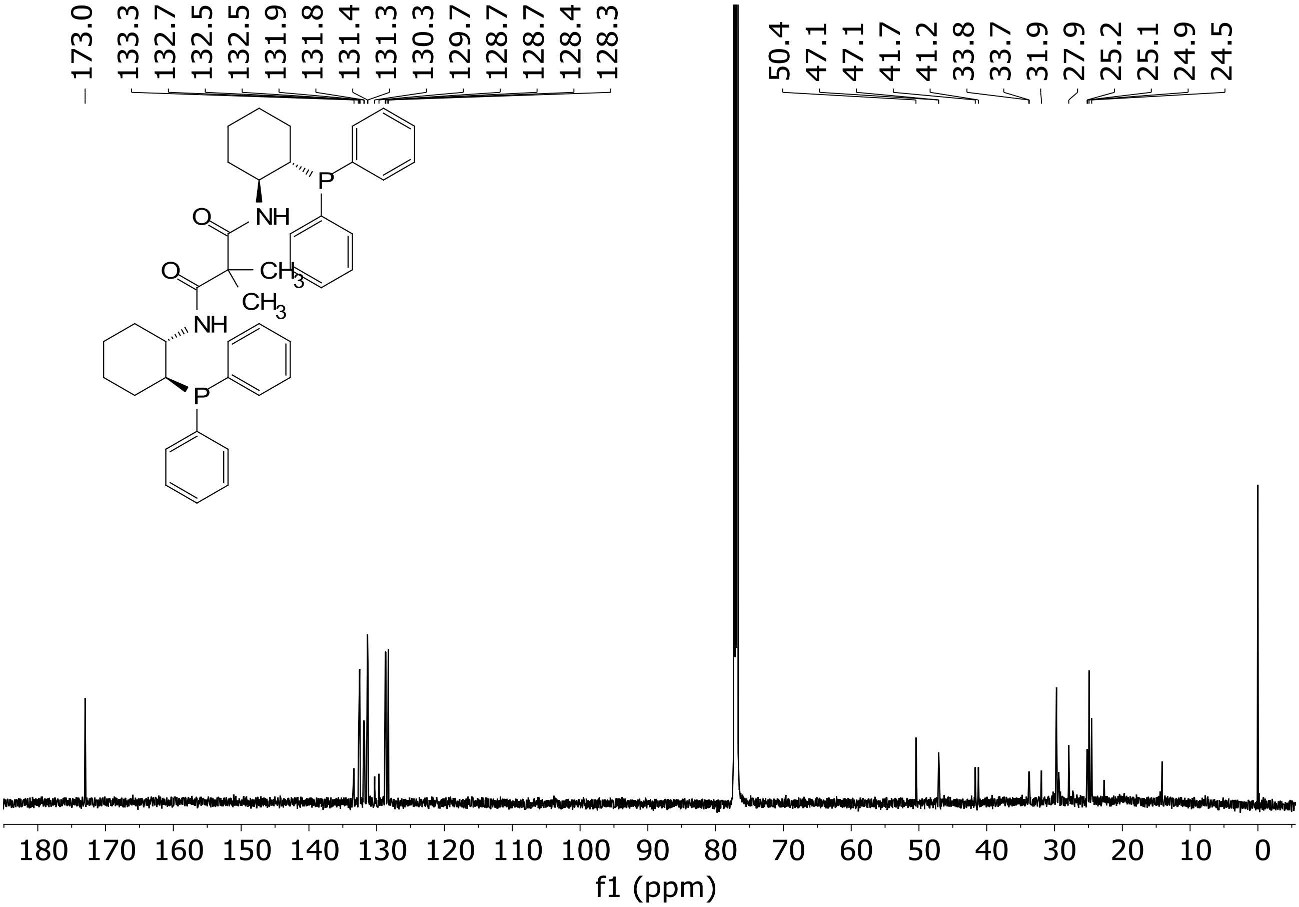

Figure S31. The ^{13}C NMR spectrum of **1c** acquired at 25 °C in $CDCl_3$ at 151 MHz

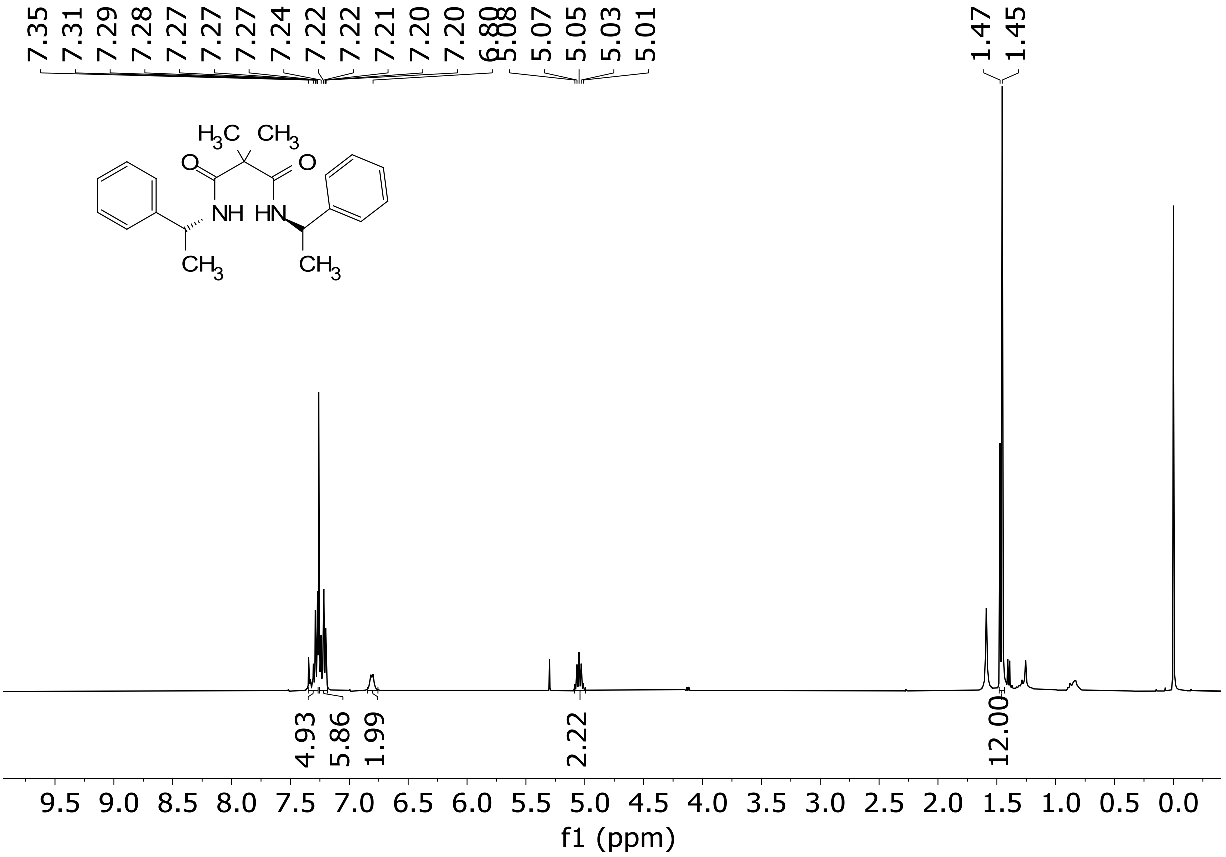

Figure S32. The ^{1}H NMR spectrum of **1d** acquired at 25 °C in $CDCl_3$ at 600 MHz.

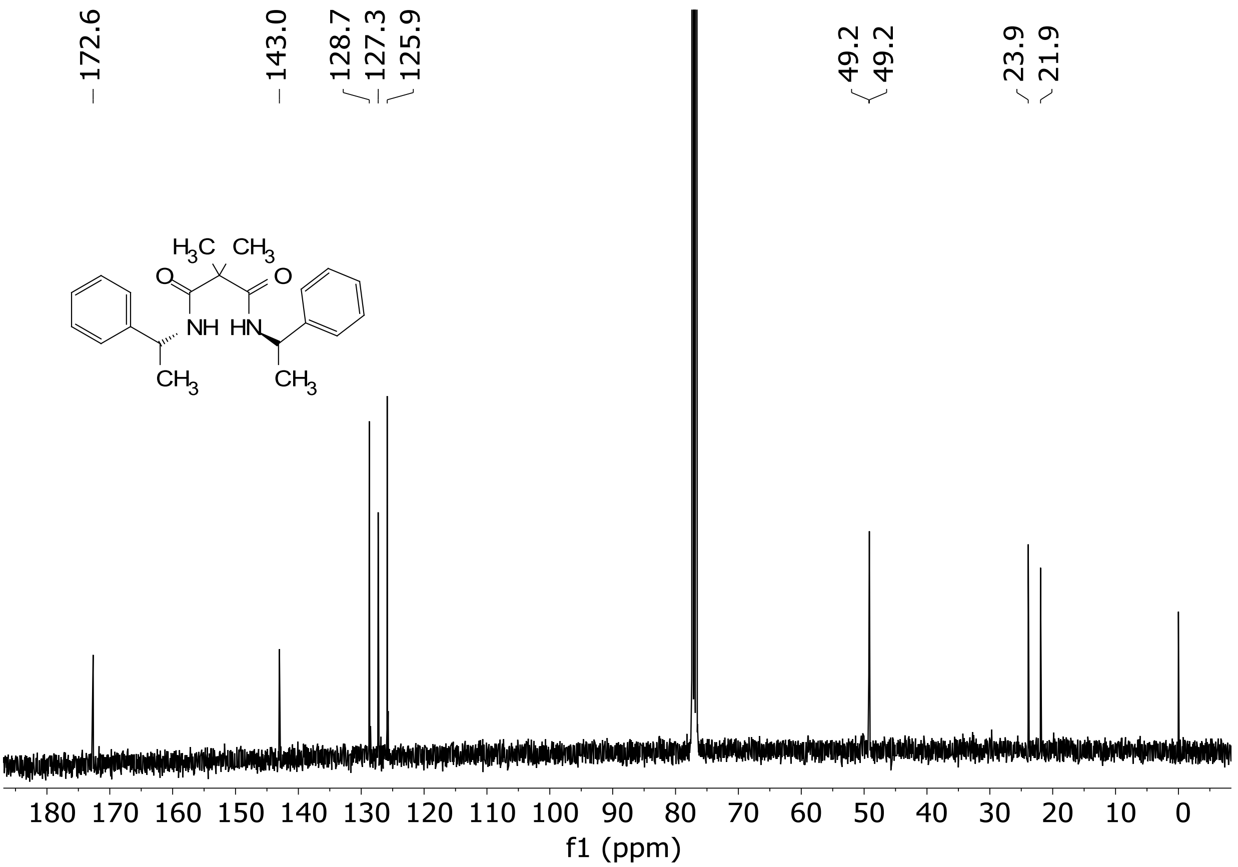

Figure S33. The ^{13}C NMR spectrum of **1d** acquired at 25 °C in $CDCl_3$ at 151 MHz.

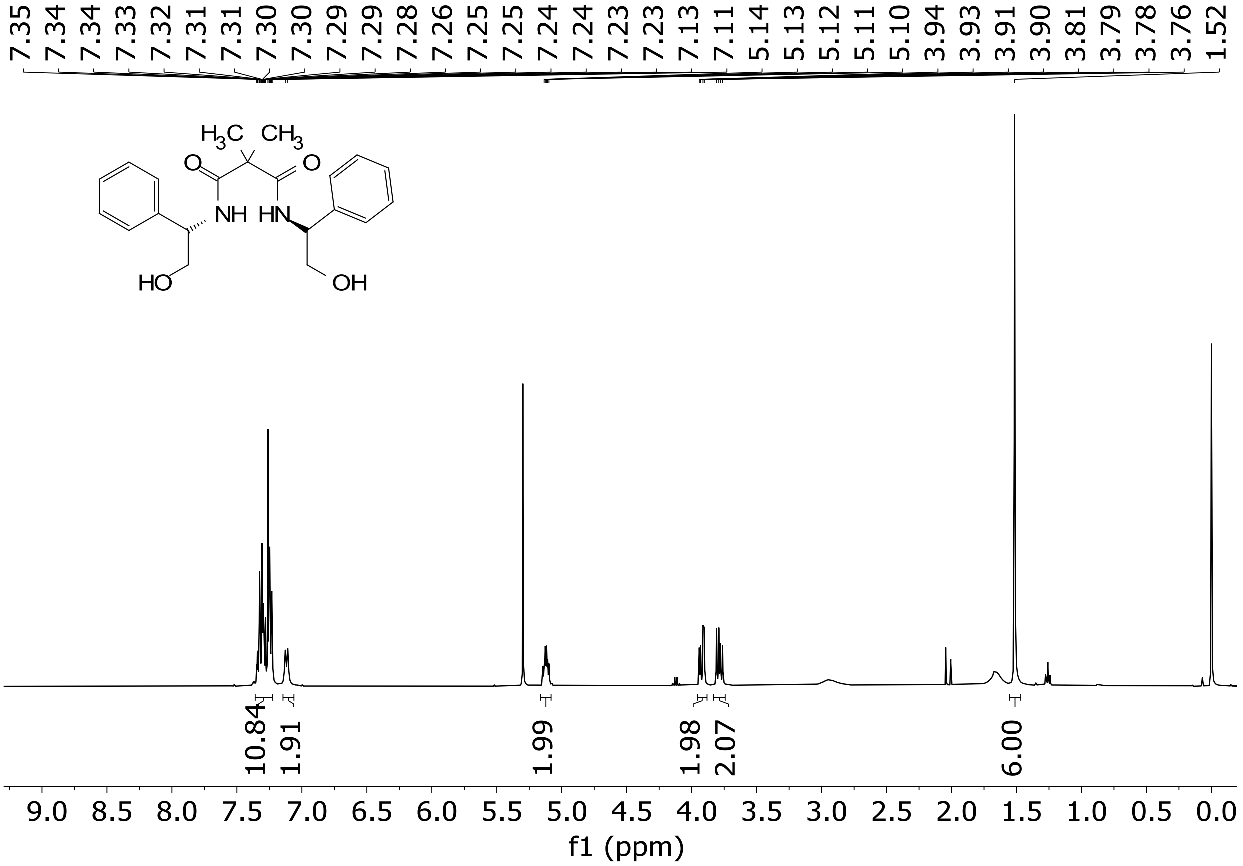

Figure S34. The ^{1}H NMR spectrum of **1e** acquired at 25 °C in $CDCl_3$ at 400 MHz.

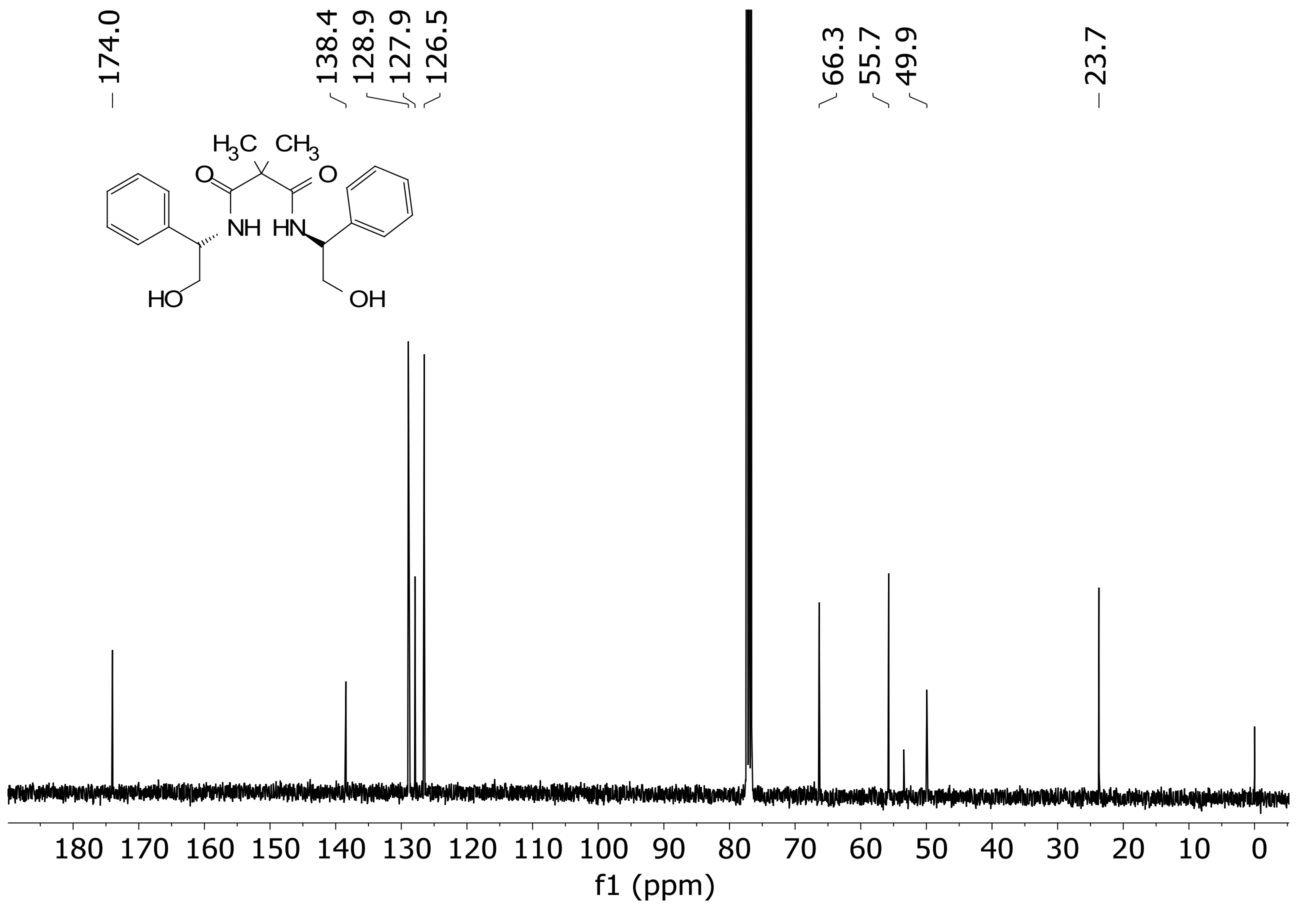

Figure S35. The ^{13}C NMR spectrum of **1e** acquired at 25 °C in $CDCl_3$ at 151 MHz.

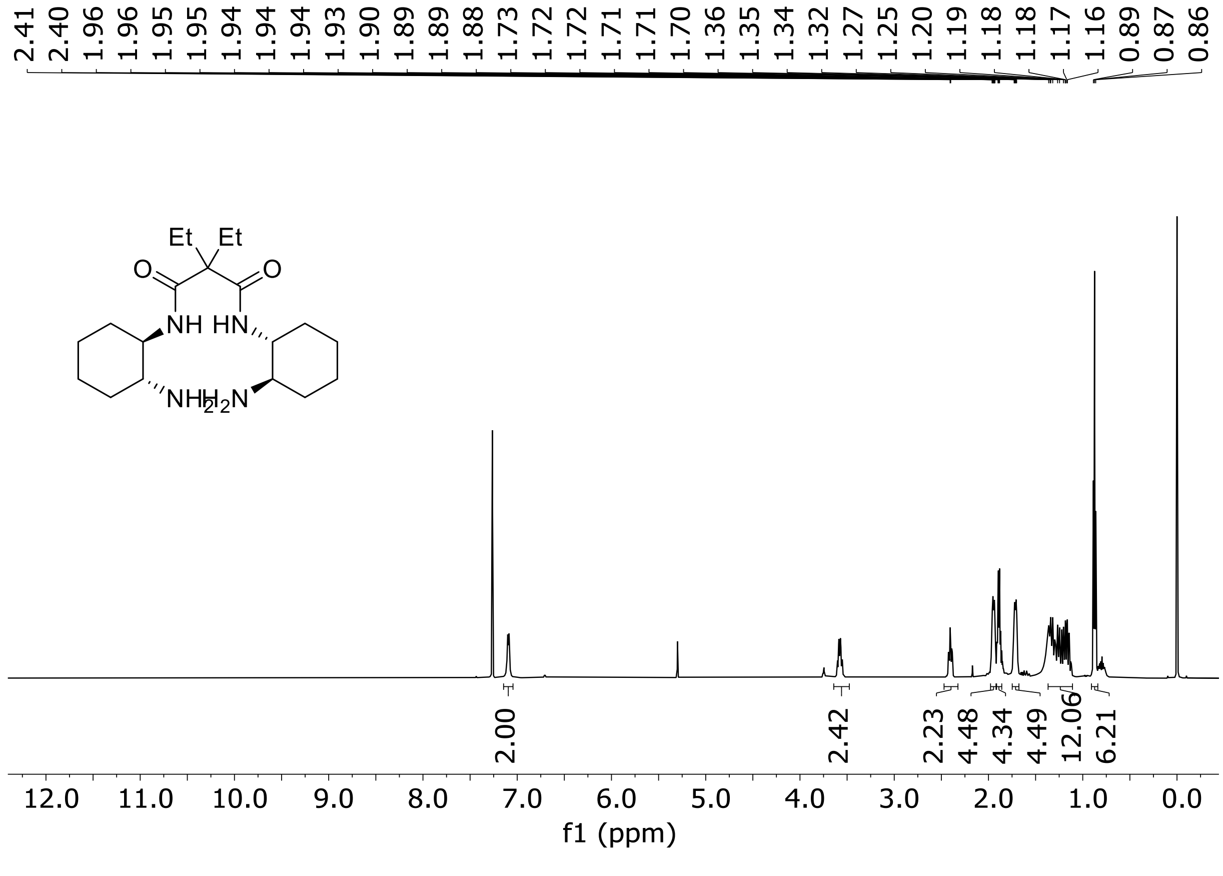

Figure S36. The ^{1}H NMR spectrum of **3a** acquired at 25 °C in $CDCl_3$ at 600 MHz.

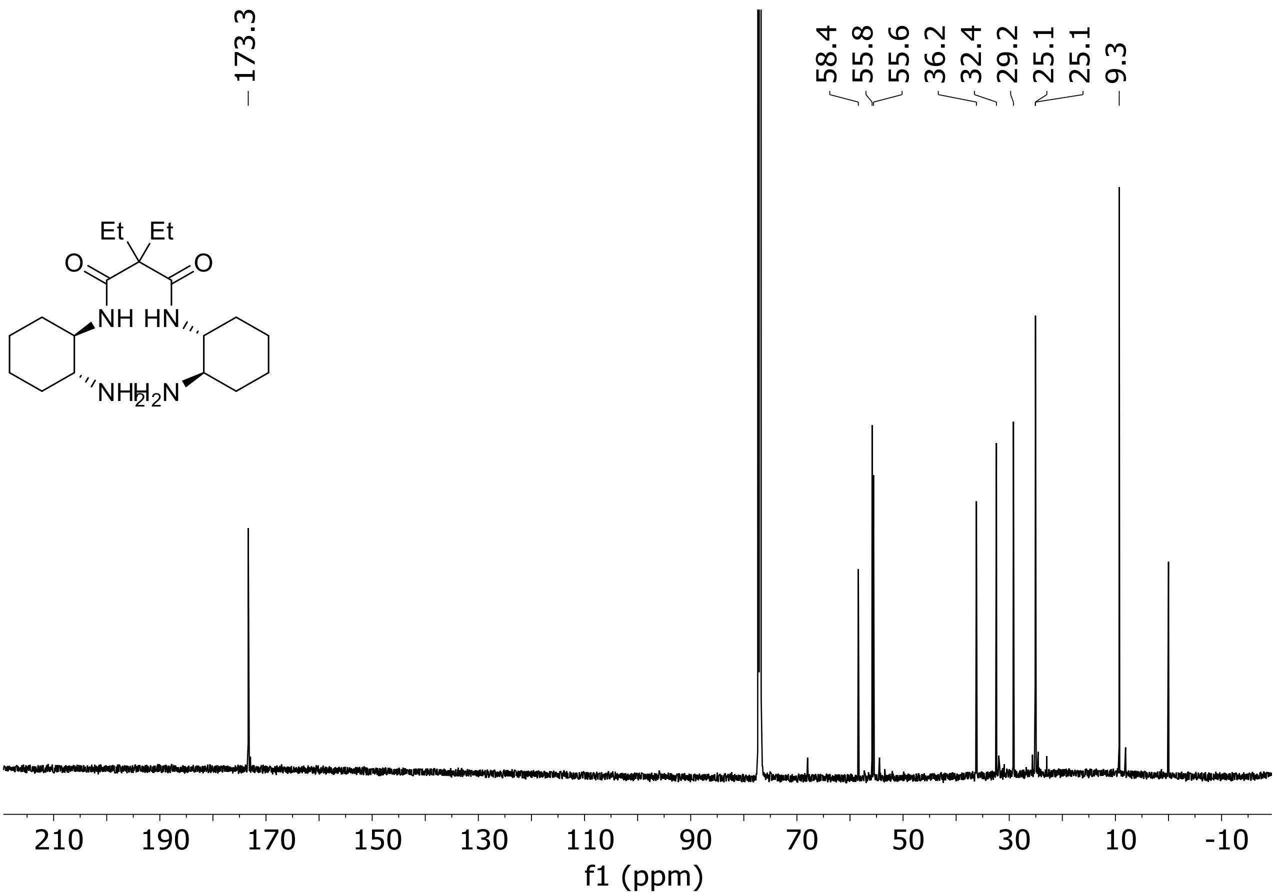

Figure S37. The ^{13}C NMR spectrum of **3a** acquired at 25 °C in $CDCl_3$ at 151 MHz

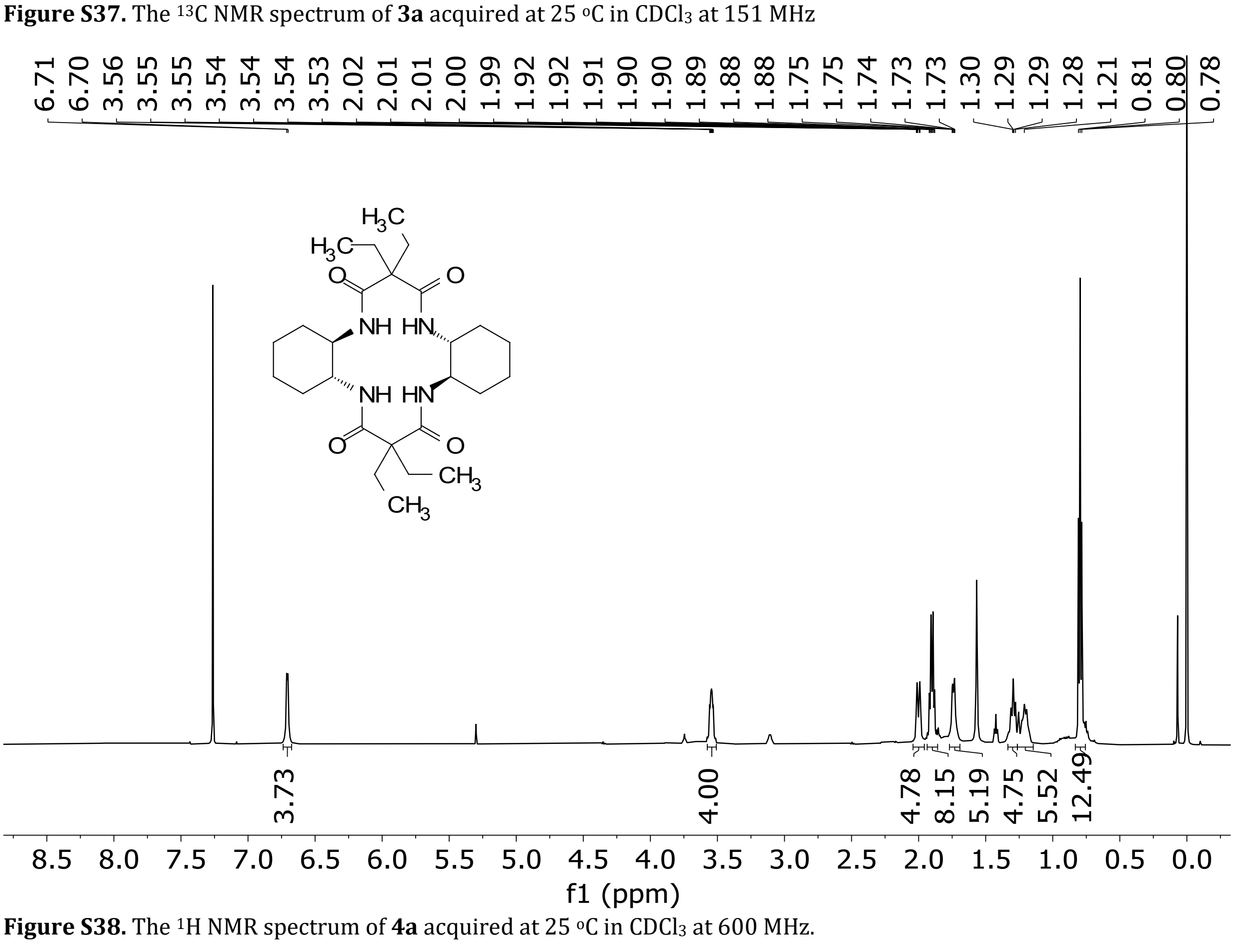

Figure S38. The ^{1}H NMR spectrum of **4a** acquired at 25 °C in $CDCl_3$ at 600 MHz.

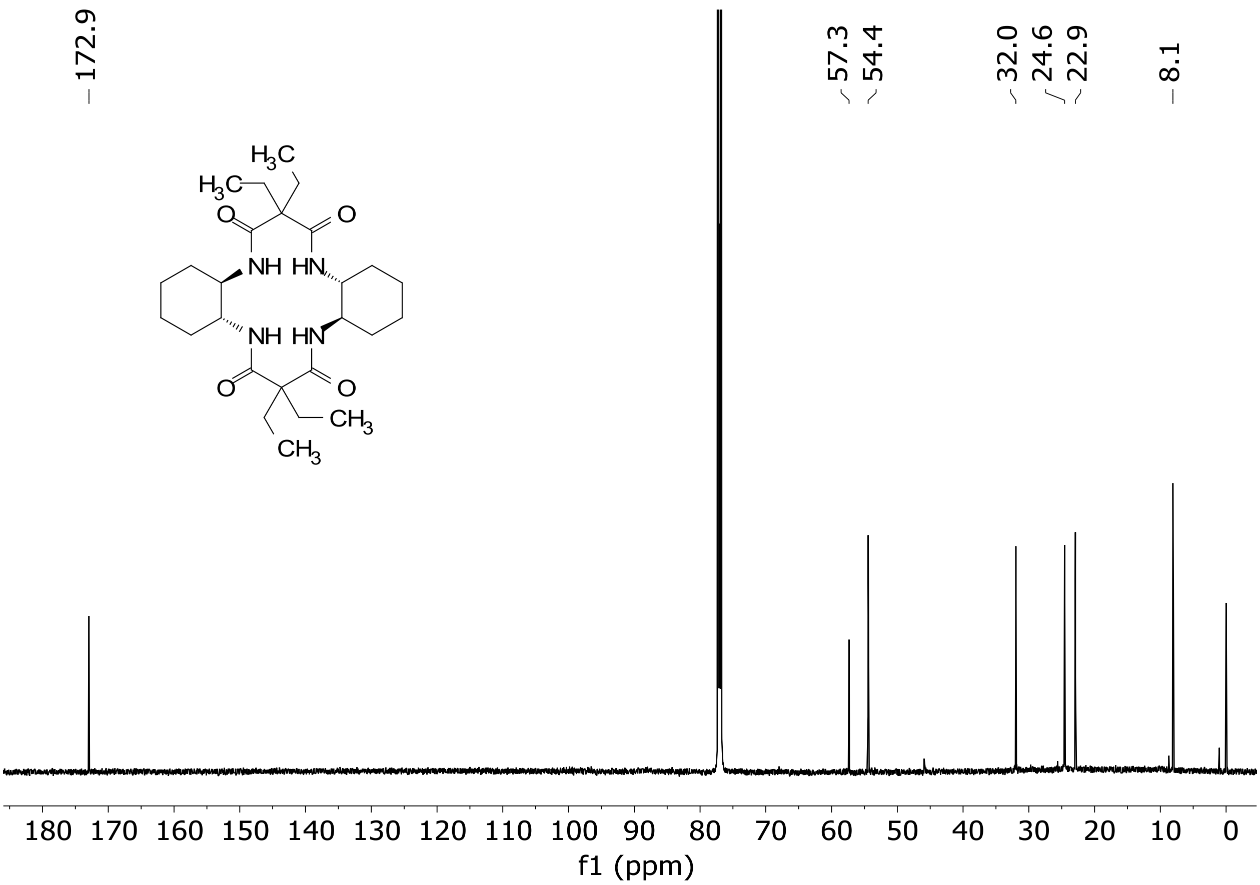

Figure S39. The ^{13}C NMR spectrum of **4a** acquired at 25 °C in $CDCl_3$ at 151 MHz.

3. References

[1.] Ciesielski, J.; Leboeuf, D.; Stern, H. A.; Frontier, A. J. *Adv. Synth. Catal.,* 2013, **355**, 2077.

[2.] Huang, G.; Cheng, C.; Ge, L.; Guo, B.; Zhao, L.; Wu, X. *Org. Lett.,* 2015, **17**, 4894-4897.

[3.] De, C. K.; Paul, A.; Emge, T. J.; Seidel, D. *Supramol. Chem.,* 2016, **28**, 168-175.

[4.] Takeda, T.; Terada, M. *J. Am. Chem. Soc.,* 2013, **135**, 15306-15309.

[5.] Corey, E. J.; Imai, N.; Zhang, H. Y. *J. Am. Chem. Soc.,* 1991, **113**, 728-729.
